# Bioinspired Cross-Linked Anionic Hydrogel Microparticles as Potential Adjuvants for Safe Vaccines: Colloidal-Chemical Characteristics, Morphology and Biological Properties

**DOI:** 10.3390/gels12080677

**Published:** 2026-07-31

**Authors:** Sebnem Ercelen Ceylan, Nataliya Mitina, Nataliya Finiuk, Bunyamin Bulkurcuoglu, Saygin San, Aise Rumeysa Mazi, Mariya Kozak, Oleh Izhyk, Iryna Petruh, Vasil M. Garamus, Khrystyna Harhay, Roman Nebesnyi, Rostyslav Stoika, Alexander Zaichenko

**Affiliations:** 1 Department of Biophysics, Hamidiye Faculty of Medicine, University of Health Sciences, Selimiye Mah. Tıbbiye Cad.38, 34668 Istanbul, Türkiye; saygin.san@sbu.edu.tr (S.S.); aiserumeysa.mazi@sbu.edu.tr (A.R.M.); 2 Department of Organic Chemistry, Institute of Chemistry, Lviv Polytechnic National University, 12, Stepan Bandera Str, 79013 Lviv, Ukraine; nataliia.y.mitina@lpnu.ua (N.M.); oleh.b.izhyk@lpnu.ua (O.I.); khrystyna.i.harhai@lpnu.ua (K.H.); roman.v.nebesnyi@lpnu.ua (R.N.); 3 Department of Regulation of Cell Proliferation and Apoptosis, Institute of Cell Biology of the National Academy of Sciences of Ukraine, 16, Drahomanov Str., 79005 Lviv, Ukraine; nataliyafiniuk@gmail.com (N.F.); stoika.rostyslav@gmail.com (R.S.); 4 Department of Medical Biology, Faculty of Medicine, İstanbul Health and Science University, Sütlüce Mah. İmrahor Cad.82, 34445 Istanbul, Türkiye; bunyamin.bulkurcuoglu@istun.edu.tr; 5 Laboratory of Molecular Biology and Genetics, Institute of Animal Biology NAAS Ukraine, 38, V. Stus str., 79034 Lviv, Ukraine; mariyarkozak@gmail.com (M.K.); irapetruh@ukr.net (I.P.); 6 Faculty of Veterinary Medicine, Stepan Gzhytskyi National University of Veterinary Medicine and Biotechnologies, Pekarska St, 50, 79000 Lviv, Ukraine; 7 Department of Optoelectronics and Information Technologies, Ivan Franko National University of Lviv, 1, Universytetska St., 79000 Lviv, Ukraine; 8 Helmholtz Zentrum Hereon, Max-Planck-Str. 1, 21502 Geesthacht, Germany; vasyl.haramus@hereon.de

**Keywords:** hydrogel microparticles, BSA immobilization, structure and colloidal-chemical characteristics, toxicity and immunogenic efficiency, model vaccine

## Abstract

Novel anionic cross-linked hydrogel-based microparticles with controlled size, functionality, porosity, and morphology were synthesized via tailored precipitation polymerization of hydrophobic and hydrophilic monomers in the presence of dimethacrylate as a cross-linking agent. The resulting hydrogel microparticles (HG5), with a size of 250 ± 50 nm and a polydispersity index of 0.079, were readily dispersible in water and formed stable suspensions at physiological pH (7.2–7.4). Their physicochemical properties were comprehensively characterized using FT-IR, NMR, DLS, SAXS, TEM, and turbidimetry. HG5 efficiently incorporated albumin as a model antigen with a loading efficiency of 95%, forming stable hydrogel–protein complexes that maintained their hydrodynamic size and colloidal stability over one month of storage. Biological evaluation demonstrated low toxicity toward pseudonormal cell lines, with cell viability remaining above 50% at concentrations up to 5 mg/mL and no detectable apoptotic effects in HEK293 cells. Human peripheral blood mononuclear cells (PBMCs) were sensitive to HG5 exposure, exhibiting an IC_50_ of 0.52 mg/mL, while showing good hemocompatibility with hemolysis below 5%. HG5 significantly enhanced immunogenicity, increasing BSA-specific antibody levels by 1.9-fold compared to the immunization without adjuvant (*p* < 0.05). These findings demonstrate the potential of HG5 as a versatile platform for protein delivery and vaccine adjuvant development.

## 1. Introduction

Functional synthetic polymers of different compositions and architectures have been proposed for use in biology, medicine, and biotechnology [1]. These include linear and branched, di- and triblock copolymers, micelles, polymer-grafted nanoparticles, polymer-decorated vesicles, polymersomes, and nano-/microporous hydrogels, each defining distinct behavior in vivo and therapeutic potential [2]. Linear polymers can facilitate drug transport across plasma membranes, depending on their physicochemical characteristics [3]. At a moderate density of charged fragments uniformly distributed along the polymer backbone, polymers promote plasma membrane association and cellular internalization. Depending on the number, type, and spatial distribution of the cationic moieties, positively charged polymers may possess significant cytotoxicity [4]. Incorporation of hydrophobic components enhances interactions with lipid bilayers, and an excessive hydrophobicity may disrupt the plasma membrane, leading to cell damage [5,6]. Consequently, the efficiency of drug delivery with linear polymer carriers depends on their molecular weight, backbone flexibility (persistence of length), hydrophilic–hydrophobic balance, and charge [2,7].

Branched polymers with optimized physicochemical profiles can cross the plasma membrane [8]. Branched copolymers have been widely applied for non-viral gene delivery, particularly for siRNA complexation via functional side chains. Hyperbranched and star-shaped polymers are internalized by cancer cells predominantly through dynamin-dependent, clathrin-mediated endocytosis [9]. Owing to their three-dimensional architecture and colloidal organization, such carriers can enable a sustained drug release. For example, colloidal systems based on branched polymers that accumulate in neuronal endosomes have demonstrated the prolonged (up to 24 h) release of the analgesic agents [10].

For using di- and triblock-based copolymer micelles, their molecular architecture plays a dual role in determining both drug loading capacity and cellular uptake. Precise control over the hydrophilic–hydrophobic balance and block lengths is an essential factor for stabilizing micelle formation and efficient encapsulation of the hydrophobic therapeutics [11,12].

Polymeric particles are efficiently internalized by antigen-presenting cells (APCs), including dendritic cells and macrophages, and can be engineered for targeted delivery through surface functionalization (e.g., carboxyl, amine, glycosidic, or oligonucleotide moieties). Such modifications promote receptor-specific interactions, enhance cellular internalization, and support cross-presentation pathways essential for robust cellular immunity. Their structural versatility also enables the incorporation of diverse antigens and their stabilization under stress conditions, including elevated temperatures [13].

Polymeric adjuvants constitute an important class of biomaterials for immunological applications. In vaccine formulations, they enhance immunogenicity by providing controlled and sustained antigen release (the depot effect) and by protecting antigens from premature degradation and nonspecific interactions with biological components [14]. A key advantage of polymeric adjuvants is the precise tunability of their physicochemical properties, including particle size (nano- or microscale), surface charge, hydrophilic–hydrophobic balance, mechanical stiffness, and degradation kinetics [15]. These parameters directly influence antigen uptake, lymphatic transport, and interactions with immune cells—features that are difficult to control with conventional adjuvants such as aluminum hydroxide and aluminum salts (Alum) [16].

Unlike traditional mineral adjuvants, which predominantly induce Th2-biased responses [17], polymeric adjuvants can be designed to stimulate balanced or tailored immune profiles, including Th1-, Th17-, and cytotoxic T-lymphocyte responses, alongside humoral immunity [18]. Many synthetic and natural polymers exhibit favorable biocompatibility and safety profiles. Owing to their capacity for prolonged antigen presentation, targeted immune modulation, and customizable design, polymeric adjuvants represent versatile platforms that are particularly valuable for vaccines targeting weakly immunogenic antigens or immunologically vulnerable populations [14,18].

Hydrogel microparticles can be engineered to interact with peptides and proteins, making them promising carriers for macromolecular drug delivery. Injectable microgel-based composites have demonstrated a sustained release of low–molecular-weight therapeutics [19]. The three-dimensional cross-linked polymer networks that constitute these gel structures provide enhanced mechanical integrity, reduce premature drug leakage, and improve pharmacokinetic profiles [20,21,22].

Ultra-low-cross-linked hydrogel microparticles have attracted growing interest due to their distinctive mechanical compliance and flow behavior. When deposited in evaporating droplets at room temperature, swollen hydrogel microparticles suppress the coffee-ring effect, and under flow conditions, they behave either as soft, deformable objects or as more rigid colloids, depending on their concentration [23,24]. These highly deformable particles can traverse capillaries narrower than their own hydrodynamic size [25].

The remarkable physicochemical properties and strong potential of hydrogel microparticles have motivated extensive work on their synthesis, bottom-up assembly, and application [26,27]. Among smart materials, hydrogel microparticles are distinguished by their high-water content, well-defined chemical and structural organization, favorable mechanical properties, and excellent biocompatibility. These features, together with an internal cross-linked network capable of encapsulating therapeutic agents, make them highly attractive for biomedical use [27]. Incorporating different functional groups enables the design of temperature- and pH-responsive microparticles for controlled drug release triggered by changes in the inflammatory microenvironment [28,29,30]. Their characteristic architecture also allows use as multifunctional carriers for the delivery and controlled release of bioactive compounds by both parenteral and oral routes [31].

Hydrogel micro- and nanostructures are three-dimensional networks formed through either covalent or non-covalent intermolecular interactions [32]. Physical cross-linking is a simple and efficient approach to nanogel synthesis. It is typically performed in aqueous media, where polymer chains are fully solvated and initially interact only weakly. Changes in temperature, pH, or ionic strength then induce inter-chain interactions and network formation. Polymers commonly used for electrostatically cross-linked nanogels include chitosan, alginate, hyaluronic acid, and poly-glutamic acid [33]. Covalent cross-linking provides enhanced stability and tunable functionality. Key synthetic approaches include free-radical polymerization in the presence of cross-linking agents [29,34,35], click chemistry (azide-alkyne) [36], thiol–maleimide/ene reactions, disulfide exchange, and carboxyl–amine coupling [37]. Microfluidic technologies based on the generation of discrete droplets enable the preparation of monodisperse hydrogel microparticles with controlled surface charge and a highly porous internal architecture, making them attractive carriers for biologically active compounds [38,39]. Precipitation and inverse emulsion polymerization are also frequently used, as both combine polymerization and cross-linking in a single step [40,41].

Previously, we generated polymer particles bearing diverse surface functional groups [42], as well as multifunctional hydrogel particles [43] that demonstrated promising performance as drug delivery carriers and as luminescent markers for recognition and labeling of pathological cells [44]. We have also prepared protein-loaded constructs with adjuvant potential that may serve as safe alternatives to conventional adjuvants such as Alum [45]. Bovine serum albumin (BSA) is widely used as a model antigen because it is immunogenic, easily purified, cost-effective, and highly stable. These properties allow immune responses and antibody production to be studied reliably, without the high costs or hazards associated with complex pathogens or with expensive recombinant proteins, which are typically available in microgram quantities (10–100 µg). BSA is moderately immunogenic in mice. It can elicit an immune response but generally requires an adjuvant to do so [46].

These findings motivated us to conduct more comprehensive investigations aimed at elucidating the colloid-chemical properties of the hydrogels and their complexes with proteins and at assessing the biocompatibility and immunogenicity of the created adjuvant systems. In the current study, we provided a basis for the rational development of a broad range of functional polymer-based adjuvants and for the identification of the most promising candidates for use in safe and effective vaccines. Such studies are essential for establishing structure–property–activity relationships (SAR) that link the composition and colloid-chemical characteristics of the hydrogel particles to their immunogenic performance.

## 2. Results and Discussion

### 2.1. Structural and Colloidal-Chemical Characteristics of HG5 and HG5-BSA Complex

The functional composition of the synthesized HG5 microparticles was confirmed using chemical analysis (Table 1), ^1^H NMR, and FTIR spectroscopy (see Supplementary Figures S1 and S2). The ^1^H NMR spectrum (Figure S1) shows characteristic signals corresponding to the polymer structure: methylene protons of the polymer backbone (–CH_2_–) at 1.6–2.0 ppm; methine protons of the backbone (–CH–) at 2.4 ppm; methyl groups (–CH_3_) in TEGDMA and GMA units at 0.7–1.2 ppm; protons of the C(O)–O–CH_2_– fragment present in TEGDMA, BA, and GMA units at 4.1 ppm; –CH–CH_2_–O– fragments of GMA at 3.4 ppm and –CH_2_–CH_2_–O– fragments of TEGDMA at 3.3 ppm.

In the FTIR spectrum (Figure S2), the bands in the 2950–2870 cm^−1^ region correspond to C–H stretching vibrations of the aliphatic methylene and methyl groups. Additional bands appear at 1709 cm^−1^ (C=O stretching), 1452 cm^−1^ (asymmetric deformation of –CH_3_), and 1254, 1244, 1169, 1163, and 1116 cm^−1^ (C–O stretching).

The hydrogel particles contain a defined fraction of carboxyl groups, which impart hydrophilicity and colloidal stability in aqueous dispersions, particularly at physiological pH (7.2–7.4). The formation of an intermolecular complex between the HG5 and BSA is mediated by electrostatic (ionic) interactions of the carboxylate groups of the microgel with the protonated amino groups of the protein. In addition, the reactive epoxy groups of GMA units in the microgel structure can interact with the nucleophilic amino groups of BSA, enabling covalent conjugation of the protein to the polymer network.

DLS measurements suggest that complex formation did not alter the hydrodynamic diameter. The increase in zeta potential (from −15 to −7 mV) reflects a reduction in the net negative surface charge, consistent with the proposed interaction between the carboxylate and amino groups (Table 1).

**Figure 1:**
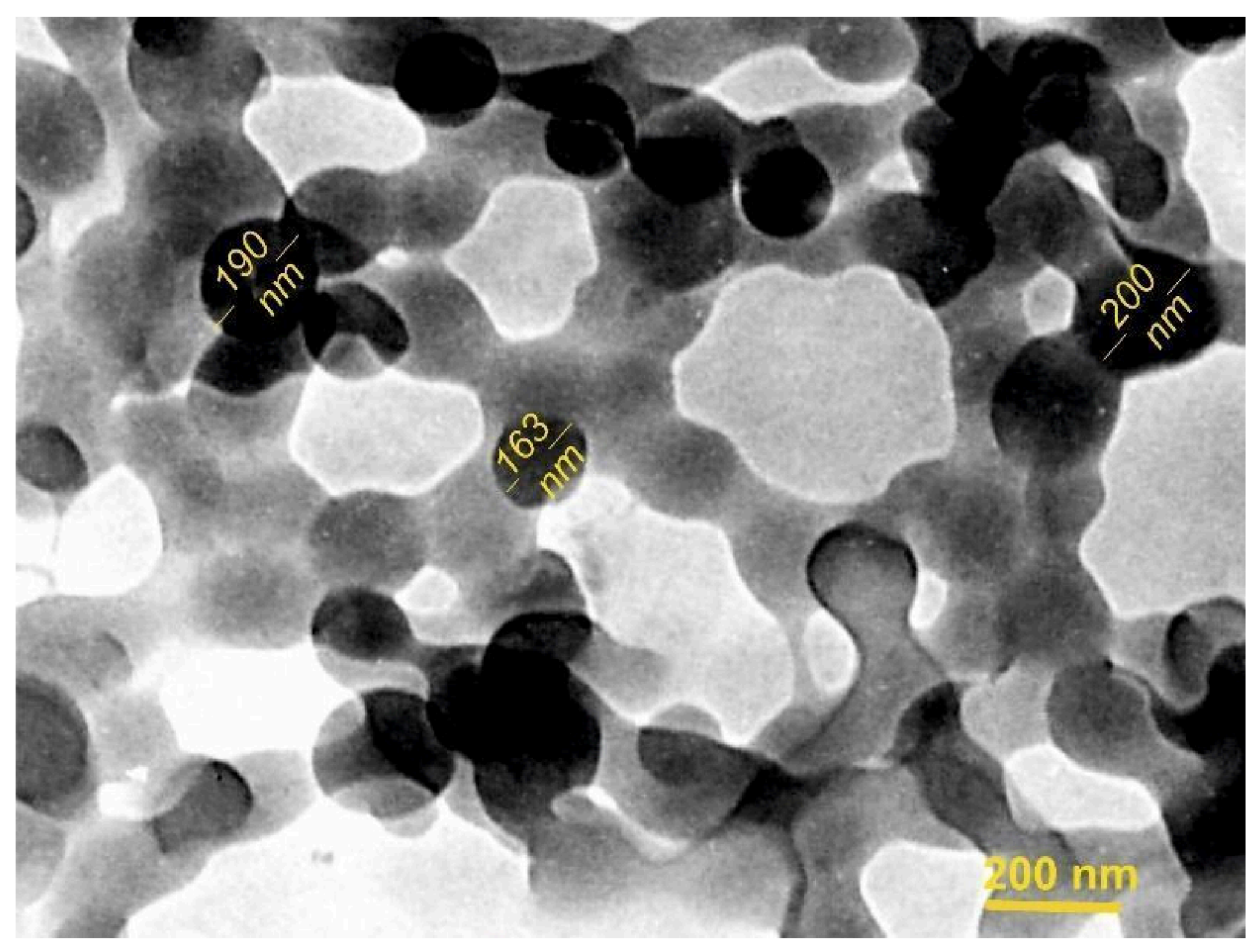
TEM image of HG5 dispersed in distilled water.

**Figure 2:**
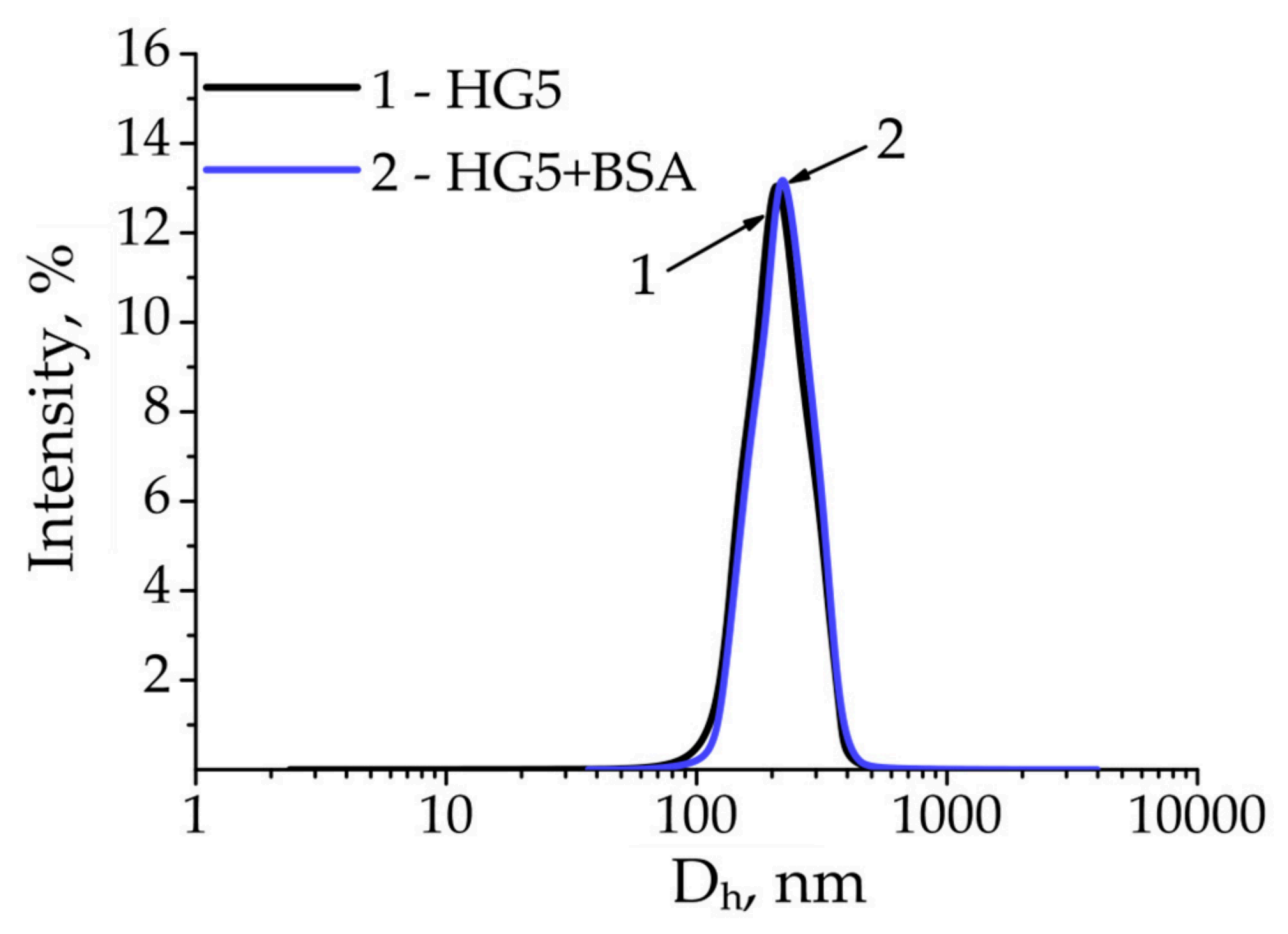
Hydrodynamic diameter of the HG5 (1) and its complex with BSA (2) (pH 7.4).

TEM imaging (Figure 1) and dynamic light scattering (DLS) (Figure 2) indicate that the hydrogel particles are predominantly of spherical shape and sizes in the range of 200–300 nm. TEM imaging also shows that these hydrophilic hydrogel particles come into close contact and appear interconnected after deposition and drying on the TEM substrate. This apparent interconnection may, however, be influenced by the sample preparation and drying process and should not necessarily be interpreted as evidence of a continuous interconnected network in the hydrated state. As expected, the hydrodynamic diameters obtained from the DLS are somewhat larger than the particle sizes estimated from TEM images, reflecting particle solvation and swelling in the water dispersion.

Particles containing ionized carboxyl groups exhibit a negative surface charge in PBS that ensures sufficient colloidal stability of the aqueous dispersions against sedimentation and aggregation.

Cross-linked HG5 was loaded with BSA that was used as a model protein for the loading study.

Figure S3 (see Supplementary Materials) indicates efficient incorporation of BSA (appr. 95%) into the hydrogel matrix and the formation of a stable HG5-BSA complex.

**Figure 3:**
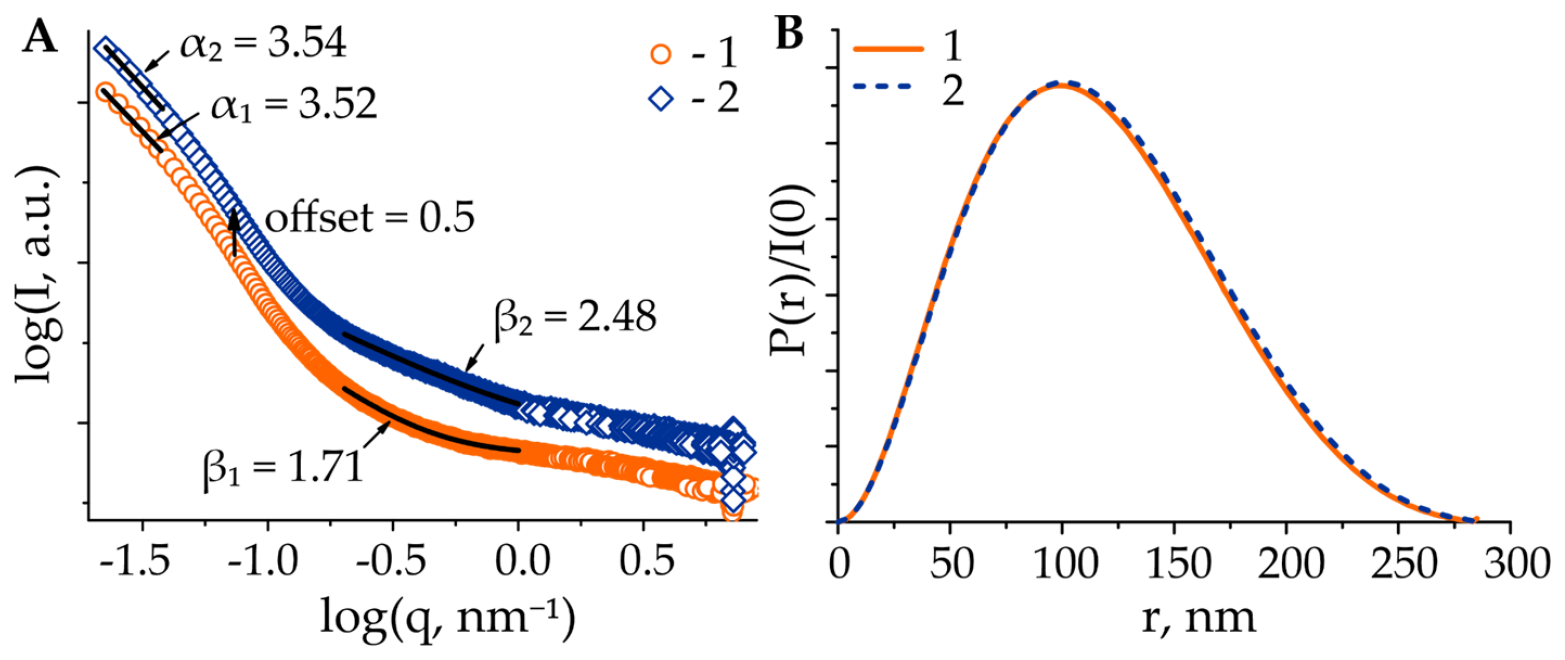
(**A**) SAXS profiles of the pristine HG5 hydrogel (1) and the corresponding HG5–BSA complex (2). (**B**) Pair distance distribution functions, P(r), calculated from the scattering curves using indirect Fourier transformation (IFT) implemented in GNOM. A vertical offset of 0.5 on the logarithmic scale was applied for clarity.

The similar particle sizes of HG5 and HG5-BSA, together with the stability of the complex (Figure 2 and Figure 3, Table S1), can be attributed to the cross-linked structure of the hydrogel network, which defines a fixed particle framework that is not substantially altered upon protein loading.

These explanations are supported by small-angle X-ray scattering (SAXS) data that provide independent confirmation of the morphology of the hydrogel particles and their complexes with proteins. SAXS profiles of the HG5 and the corresponding HG5–BSA complexes were used to determine the average size, maximum particle dimension (D_max_), and approximate particle shape before and after electrostatic binding between HG5 and BSA, as well as to probe changes in the internal structure of the hydrogel. The experimental scattering curves and the extracted parameters are presented in Figure 3 and Table S1 (see Supplementary Materials).

Comparison of the buffer-extracted SAXS profiles (Figure 3A) of pristine HG5 and the corresponding supramolecular HG5–BSA assemblies showed no significant changes in the low-q region, indicating that the average size of the hydrogel particles is not affected by BSA loading. The radii of gyration (R_g_) obtained from the Guinier approximation and from GNOM-based indirect Fourier transformation (IFT) [47] are in reasonable agreement, yielding values of 81 nm and 91 nm, respectively (Table S1). However, these values should be interpreted with caution, since the minimum measured scattering vector (q_min_ ≈ 0.022 nm^−1^) corresponds to an upper resolvable size of approximately 45 nm, so the R_g_ of such large particles may be underestimated. This conclusion is supported by the results of DLS measurements.

The nearly symmetrical shape of the pair-distance distribution functions P(r) (Figure 3B), calculated from I(q) over the q-range of 0.023 ÷ 1.0 nm^−1^, indicates that both the hydrogels and the corresponding complexes exhibit an approximately globular particle morphology. These almost identical P(r) curves suggest that there are no detectable changes in particle shape or maximum particle dimension (D_max_) upon BSA loading, which points to BSA being incorporated without major rearrangement or disruption of the hydrogel matrix and without aggregation of BSA.

Complexation of the HG5 with BSA results in a pronounced change in the Porod exponent β, from 1.71 to 2.48, indicating a denser internal structure of the hydrogels after BSA uptake. This change can be attributed to the characteristic size of BSA (~7 nm), as the investigated q region of 0.2–1 nm^−1^ corresponds to a real-space size scale (2π/q) of approximately 6–31 nm.

The formation of the HG5–BSA complex was confirmed by shifts in the FT-IR bands corresponding to the functional groups of both the anionic hydrogel particles and BSA (Figure S2B, Supplementary Materials). As shown in Figure S2B, the C=O stretching band of the acrylate groups in HG5 shifted from 1704 to 1710 cm^−1^ upon complex formation. In addition, the characteristic amide bands of BSA were shifted: the amide I band moved from 1635 to 1650 cm^−1^, while the amide II band shifted from 1523 to 1534 cm^−1^, corresponding to changes of 15 and 11 cm^−1^, respectively. These spectral shifts indicate interactions between the functional groups of the hydrogel particles and BSA, confirming the formation of the HG5–BSA complex.

**Figure 4:**
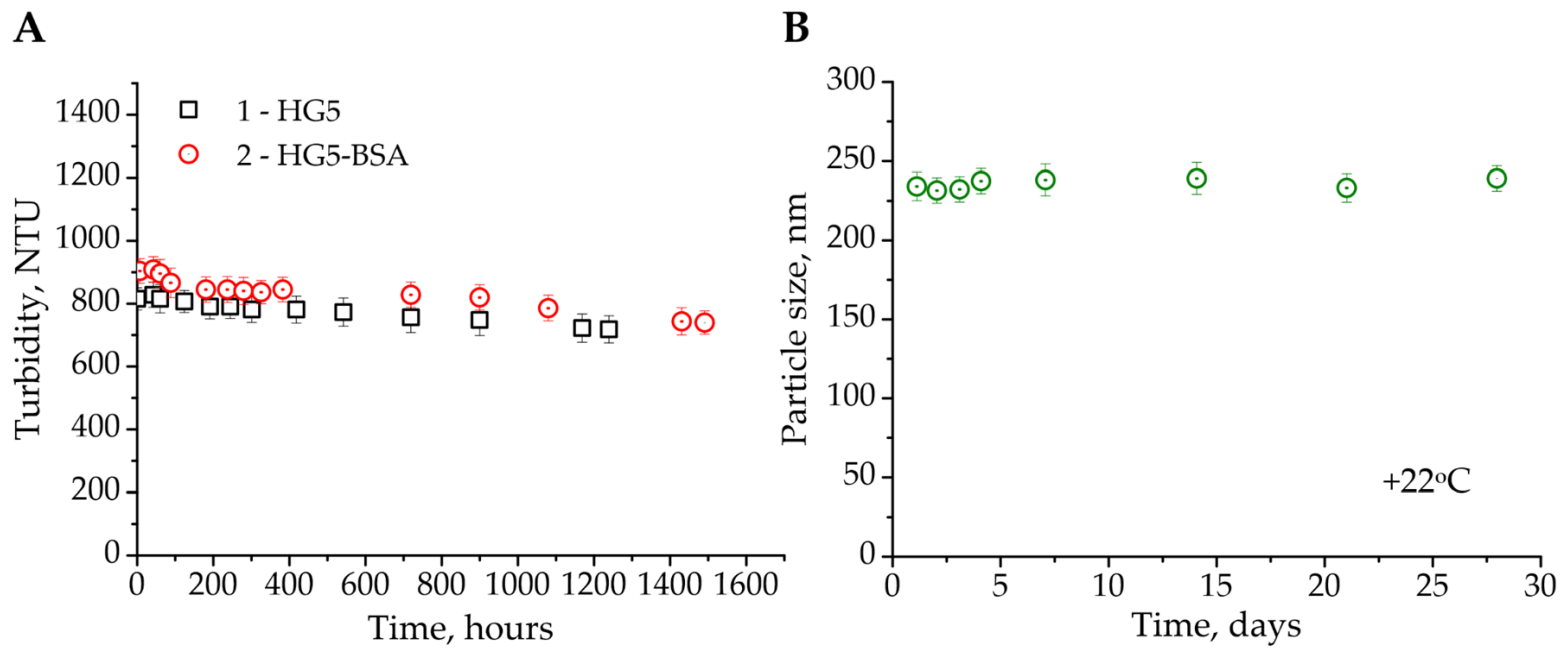
Measurement of the turbidity of aqueous dispersions of hydrogel particles HG5 (1) and its complex with BSA (2) (concentration of hydrogel particles = 0.2 mg/mL, concentration of BSA = 0.05 mg/mL) (**A**) and time-dependent particle size of the HG5-BSA complex at 22 °C (**B**) (concentration of hydrogel particles = 40 mg/mL, concentration of BSA = 10 mg/mL). Black squares represent HG5, while red and green circles represent the HG5–BSA complexes.

The stability of aqueous dispersions of hydrogel particles and their complexes with BSA was evaluated by the turbidimetric method (Figure 4A) and by monitoring temporal changes in particle size (Figure 4B). Both the hydrogel particle dispersions and their BSA-loaded complexes exhibited high stability. Notably, the hydrodynamic diameter of the BSA-loaded hydrogel particles remained unchanged throughout the one-month observation period, indicating high colloidal stability of these complexes.

The stability of the aqueous complexes arises both from the microparticle size, which satisfies the conditions for Brownian motion, and from the high entropic contribution to the Gibbs free energy of mixing between the polymer network of HG5 and water as the solvent [48]. Consequently, the crosslinked structure of the HG5 microparticles contributes to the elastic term, which opposes the entropic driving force of mixing. This balance results in optimal swelling of HG5 that satisfies both requirements: high loading capacity and dispersion stability [49]. The intermediate values of the swelling coefficient (Q = 13.2 ± 0.5) and the expansion factor (α = 2.3 ± 0.03) obtained from swelling measurements thus confirm porous hydrogel structure and indicate favorable conditions for BSA loading and stability of the corresponding complexes.

**Figure 5:**
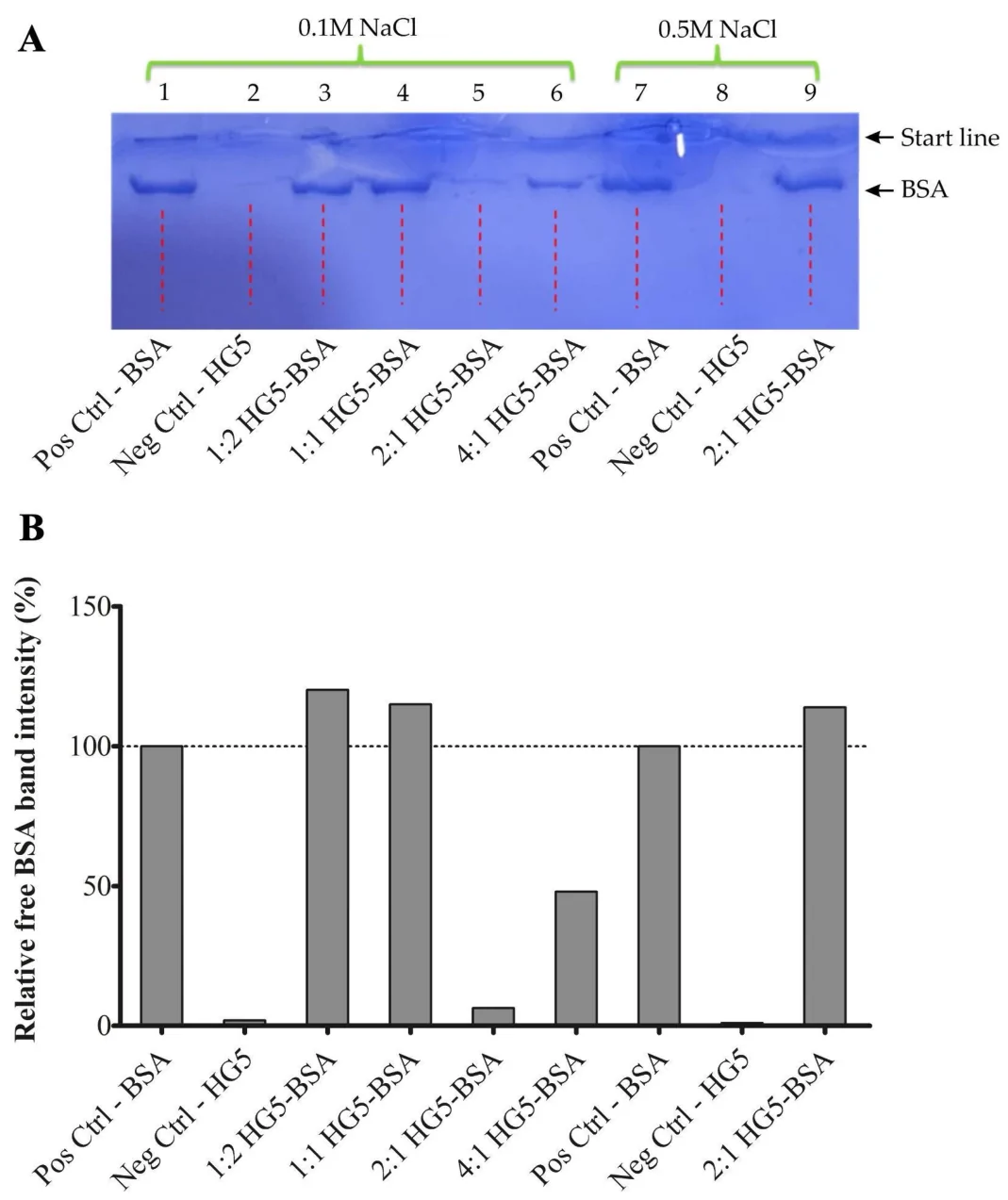
Electrophoretic study (SDS-PAGE) of the effect of NaCl concentration and HG5-BSA mass ratio on complex formation (**A**) Representative SDS-PAGE gel showing the electrophoretic profiles of the positive controls (BSA), negative control (HG5), and HG5–BSA complex samples. Arrows indicate the origin of electrophoresis and the migration position of free BSA. (**B**) Densitometric analysis of the free BSA bands quantified using ImageJ (version 1.44p; National Institutes of Health, Bethesda, MD, USA) and normalized to the free BSA control (100%).

As shown in Figure 5, the highest loading efficiency was observed in the lane corresponding to the 2:1 (HG5–BSA) formulation. In the SDS-PAGE analysis, samples containing HG5 exhibited a marked reduction in the intensity of the free BSA band compared with the free BSA control, indicating increased retention of BSA within the HG5 matrix. Densitometric analysis, normalized to the free BSA control Figure 5B (100%), confirmed that the 2:1 (HG5-BSA) formulation exhibited the lowest relative free BSA band intensity, consistent with the highest loading efficiency of BSA. Since the densitometric analysis was performed on a single representative SDS-PAGE gel (*n* = 1), these results should be interpreted as semi-quantitative and supportive of the qualitative electrophoretic findings. Importantly, the formation of the HG5–BSA complex was independently corroborated by FT-IR spectroscopy (Figure S2B) and SAXS analysis (Figure 3), while the stability of the complexes was further supported by DLS and turbidimetric measurements (Figure 4).

**Figure 6:**
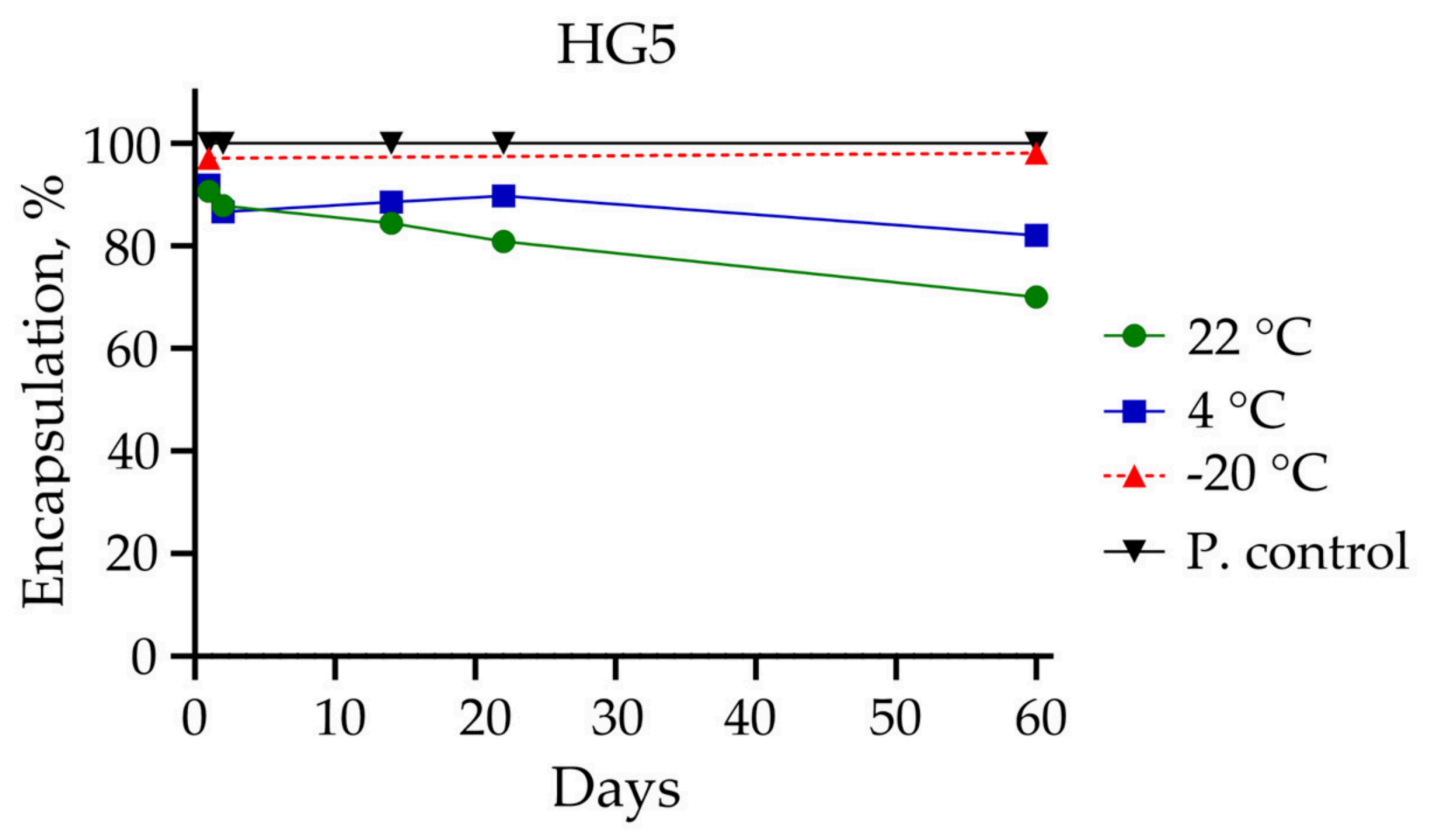
Time-dependent encapsulation profile of the HG5–BSA complex under different storage conditions.

Figure 6 demonstrates the time-dependent encapsulation profile of the HG5–BSA complex under different storage conditions. The HG5–BSA complex maintained a high encapsulation percentage throughout the storage period, with temperature-dependent differences observed. The encapsulation percentage remained almost unchanged under −20 °C storage conditions, while a gradual decrease was observed at 4 °C and 22 °C. After 60 days, approximately 80% and 70% of the initial BSA encapsulation level were maintained at 4 °C and 22 °C, respectively. These findings indicate that lower storage temperatures improve the preservation of the HG5–BSA complex and support the storage stability of the hydrogel-based antigen delivery system.

### 2.2. Study of HG5 Cytotoxicity

Aluminum hydroxide and aluminum phosphate are widely used in vaccines as enhancers of immune responses. However, while generally considered safe, studies have indicated their toxic effects under certain conditions [50]. Hydrogels generally exhibit high biocompatibility and low cytotoxicity for human cell lines, making them suitable for biomedical applications. However, some cross-linked functional hydrogel particles can cause slight cytotoxicity by decreasing cell viability [51].

The biocompatibility of HG5 was studied in vitro through evaluating its effect on the metabolic activity (MTT assay) of normal human PBMCs, human pseudonormal keratinocytes of the HaCaT line, fibroblasts of the BJ line, bronchial epithelial cells of the BEAS-2B line, human leukemia Jurkat T-cells and cells of the K562 line, and colorectal carcinoma cells of the HT-29 line. The ISO 10993-5:2009 [52] states that a material is considered non-cytotoxic if the viability (survival) of cells after exposure to it exceeds 70% relative to the control group. This threshold is generally accepted when assessing the biocompatibility of medical devices using in vitro tests, particularly MTT, XTT, NRU, and other methods.

**Figure 7:**
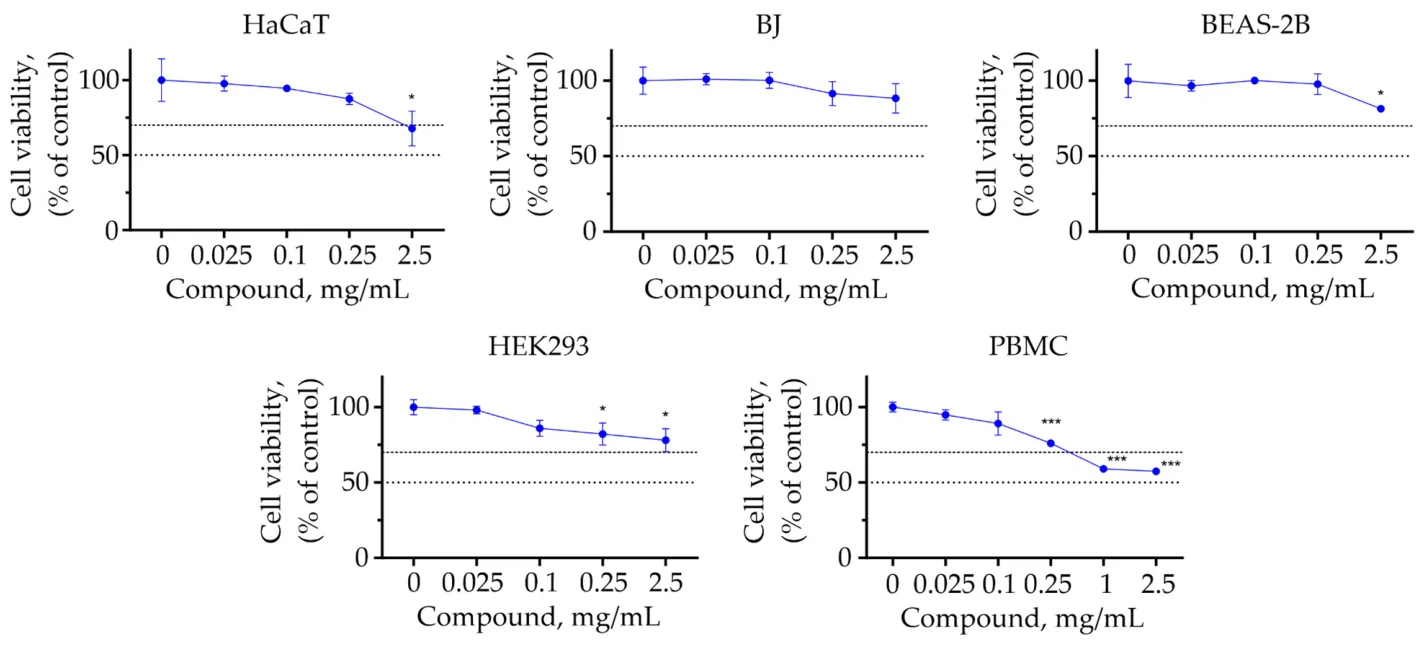
The viability measurement of human non-tumor keratinocytes of HaCaT line, BJ fibroblasts, BEAS-2B bronchial epithelial cells, HEK293 embryonic kidney cells, and PBMC from healthy human donors at cell treatment for 72 h with the HG5. The MTT assay results were expressed as a percentage relative to the control and reported as M ± SD, with *n* = 4. Horizontal lines are drawn at the 70% and 50% levels. * *p* < 0.05; *** *p* < 0.001 compared to the control.

HG5 demonstrated low toxicity towards human non-normal cells of the HaCaT line (keratinocytes), BJ line (fibroblasts), BEAS-2B line (bronchial epithelial cells), and HEK293 cell line (embryonic kidney) (Figure 7). HG5 did not reach the IC50 value of 2.5 mg/mL for the examined non-normal cells. HG5 decreased the HaCaT cell viability to 70% at the dose of 2.26 mg/mL. Thus, the dose of HG5 < 2.26 mg/mL could be identified as non-toxic for HaCaT cells. HG5 at the dose of 2.5 mg/mL could be identified as non-toxic for BJ, BEAS-2B, and HEK293 cells.

HG5 exhibited greater cytotoxicity towards human peripheral blood mononuclear cells (PBMCs) than towards human pseudonormal cells of HaCaT, BJ, HEK293, and BEAS-2B lines. The IC_50_ of HG5 was >2.5 mg/mL. This hydrogel at the dose of 0.52 mg/mL decreased the viability of isolated PBMC to 70% of the control (untreated cells) (Figure S4, see Supplementary Materials).

The HG5 hydrogel at 5 mg/mL demonstrated low toxicity towards human leukemia Jurkat and K562 cells, as well as colorectal carcinoma HT-29 cells. At that dose, HG5 reduced the percentage of viable Jurkat, K562, and HT-29 cells to 81.37 ± 0.99%, 97.66 ± 3.18, and 82.87 ± 2.07 of the control, respectively. Thus, HG5 could be identified as non-toxic for Jurkat, K562, and HT-29 cells.

**Figure 8:**
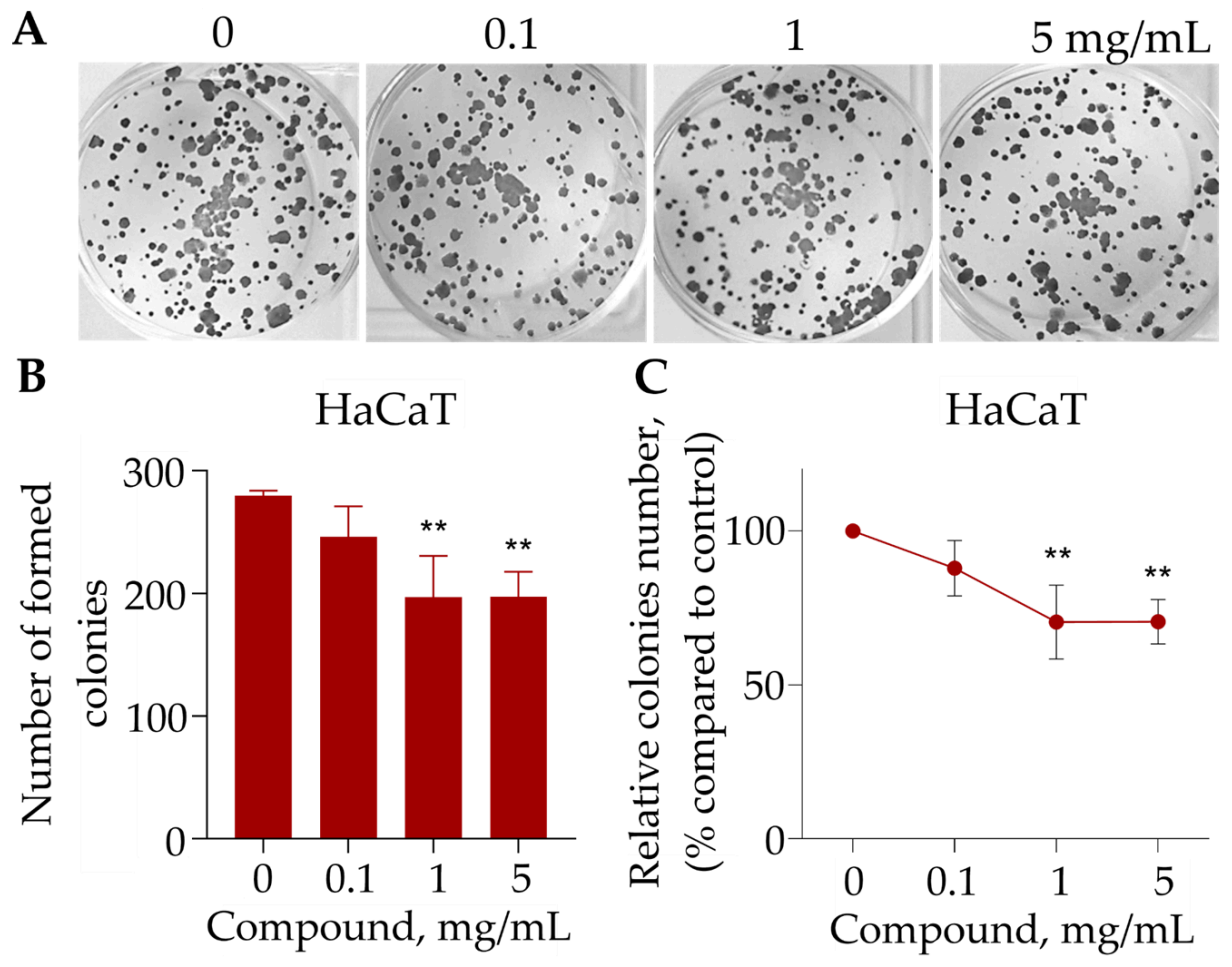
The results of the measurement of the effect of HG5 hydrogel on the clonogenic ability of human keratinocytes of HaCaT line under 14 days of cell exposure to this hydrogel: (**A**)—the representative pictures of the formed colonies; (**B**,**C**)—relative numbers of the colonies formed by the treated cells. Data are expressed as M ± SD of three independent experiments (*n* = 3) in triplicate. ** *p* < 0.01 compared to control cells.

A colony formation assay was conducted to identify the long-term inhibitory effect of the HG5 hydrogel on the HaCaT line. The hydrogel microparticles inhibited the colony formation of HaCaT cells to 87.98 ± 8.98% at 0.1 mg/mL, to 70.48 ± 12.00% at 1 mg/mL, and to 70.60 ± 7.23% at 5 mg/mL (Figure 8).

### 2.3. HG5 Effect on DNA Stability and Apoptosis in HEK293 Cells

It was reported that Al-containing adjuvants significantly affected the liver, lung, heart, and kidney functions. Several mechanisms have been proposed to explain Al-containing adjuvant-induced cytotoxic effects. This toxicity likely arises through excessive generation of reactive oxygen species (ROS), including hydrogen peroxide, hydroxyl radicals, nitric oxide, and superoxide anion, which drive oxidative stress and lipid peroxidation. Genotoxic and inflammatory pathways may also contribute to this action. Alum hydroxide nanoparticles have been shown to bind proteins and enzymes and to boost ROS output, disrupting the normal functioning of antioxidant defense systems [50]. Elevated ROS levels, in turn, activate enzymatic antioxidants (catalase, superoxide dismutase, glutathione peroxidase, and glutathione reductase) along with non-enzymatic markers (total antioxidant capacity, total oxidative status, and malondialdehyde content) [53].

These findings underscore the importance of carefully considering dosage and administration methods in vaccine formulations to minimize the potential biological risks.

**Figure 9:**
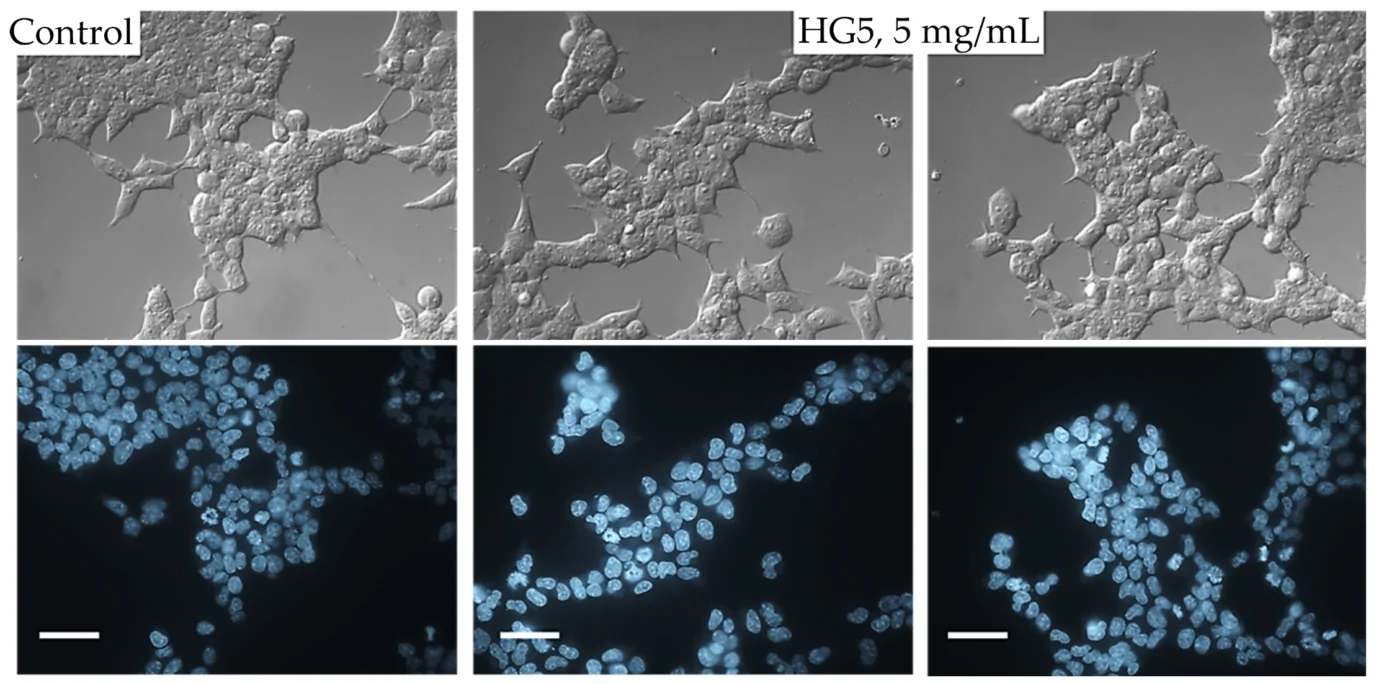
Differential interference contrast (DIC) microscopy and fluorescent images of HEK293 cells treated for 72 h with the HG5 at a 5 mg/mL dose. Top row—DIC images; bottom row—fluorescent images of treated cells stained with Hoechst 33342. Scale bar: 20 μm.

HG5 (5 mg/mL) did not induce morphological changes in HEK293 cells, such as apoptotic body formation, plasma membrane blebbing, chromatin condensation, or DNA fragmentation (Figure 9).

DNA represents a key molecular target for anti-cancer drugs, such as doxorubicin, 5-fluorouracil, cisplatin, gemcitabine, and etoposide. Damage to DNA can trigger cell cycle arrest and also lead to cell death. To quantify the extent of DNA fragmentation under HG5 treatment for 72 h, the diphenylamine assay was employed [54]. The treatment of HEK293 cells with HG5 did not cause significant fragmentation of nuclear DNA in HEK293 cells (11.21 ± 5.91%, Figure S5; see Supplementary Materials), compared with 8.20 ± 3.29% in non-treated control cells.

We conducted a comet assay to assess single- and double-strand DNA breaks, alkali-labile sites, and other lesions in HEK293 cells upon treatment with HG5. HG5 did not induce the appearance of tail DNA in the treated HEK293 cells (Figure S6, see Supplementary Materials). The percentage of tail DNA was 6.09 ± 1.37% in HG5-treated cells, compared with 4.81 ± 1.17% in the control cells.

**Figure 10:**
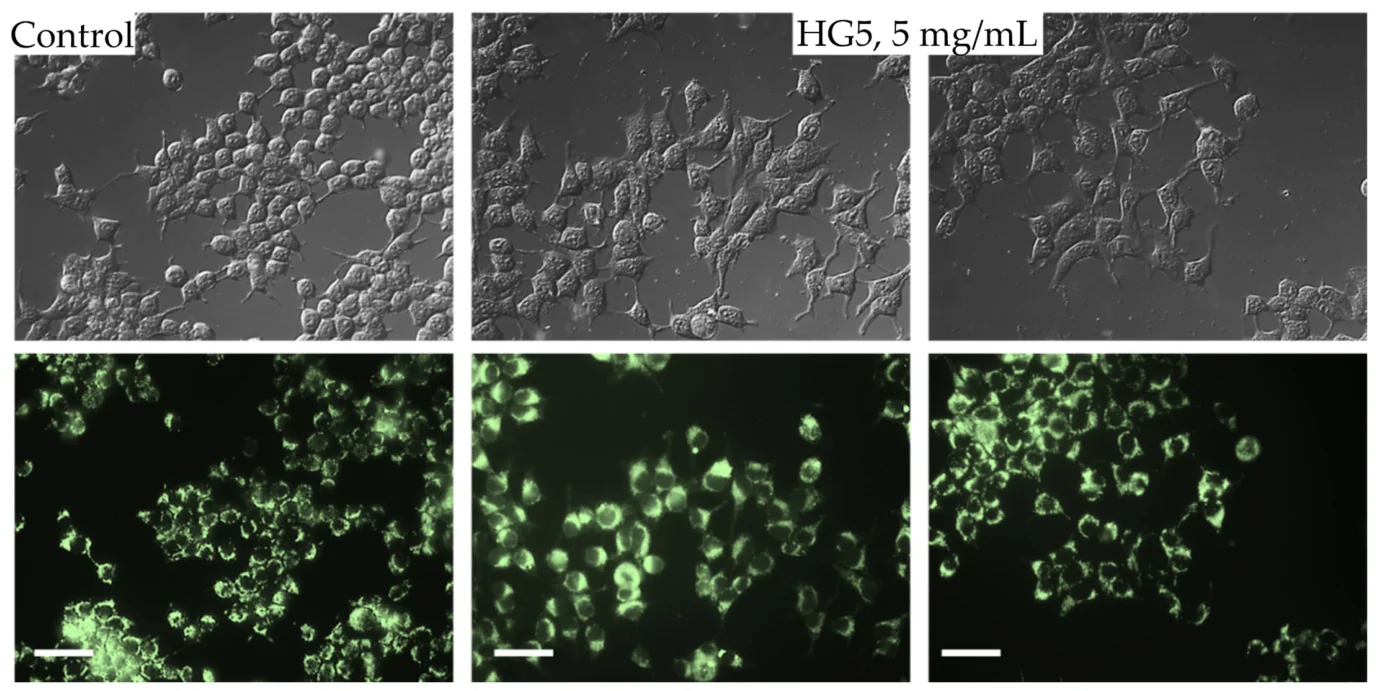
Micrographs of HEK293 cells treated for 72 h with HG5 hydrogel at 5 mg/mL dose. Top row—DIC images of treated cells. Bottom row—fluorescent images of treated cells stained with Rhodamine 123. Scale bar: 20 μm.

The treatment of HEK293 cells with HG5 (5 mg/mL) did not lead to significant changes in Rhodamine 123 fluorescence (an indicator of the level of the mitochondrial membrane potential) in these cells (Figure 10).

In conclusion, the HG5 hydrogel exhibited a low growth-inhibitory effect on the viability of pseudonormal HEK293 cells. It did not induce pro-apoptotic morphological changes in these cells. In addition, HG5 did not reduce mitochondrial membrane potential in the treated HEK293 cells.

### 2.4. Hemolytic Activity

**Figure 11:**
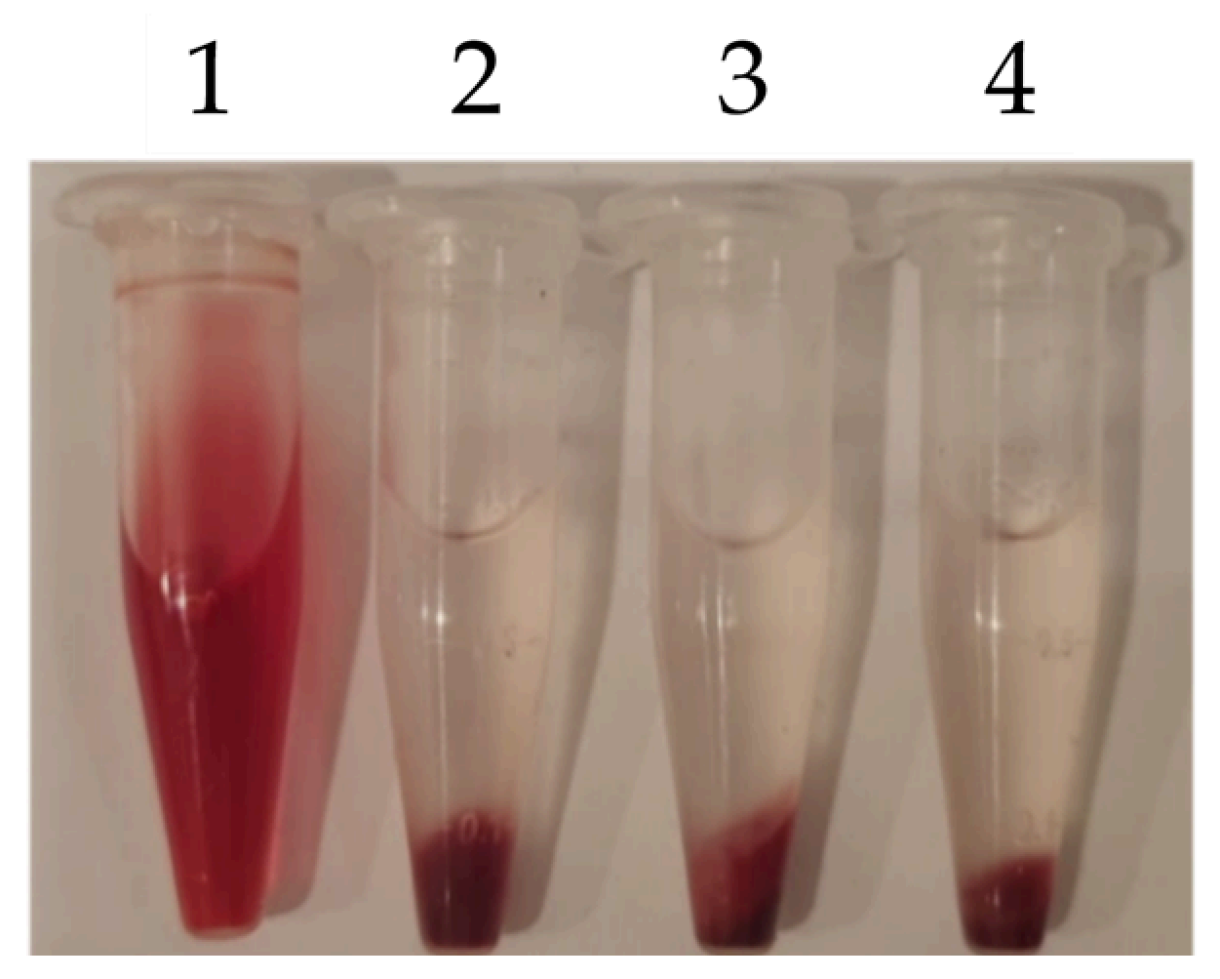
Analysis of the hemolytic activity of HG5 hydrogel. 1—Triton X-100 (concentration 0.1%), 2—1× PBS, hydrogel concentrations: 3—25 mg/mL, 4—0.25 mg/mL.

The hemolysis assay showed that the HG5 hydrogel possessed a negative zeta potential and did not induce visible red discoloration of the medium, indicating the absence of erythrocyte damage and hemoglobin release. Quantitative measurements revealed that the hemolysis level was below 5%, confirming the hemocompatibility of the HG5 hydrogel (Figure 11).

### 2.5. Immunological Studies of HG5

**Figure 12:**
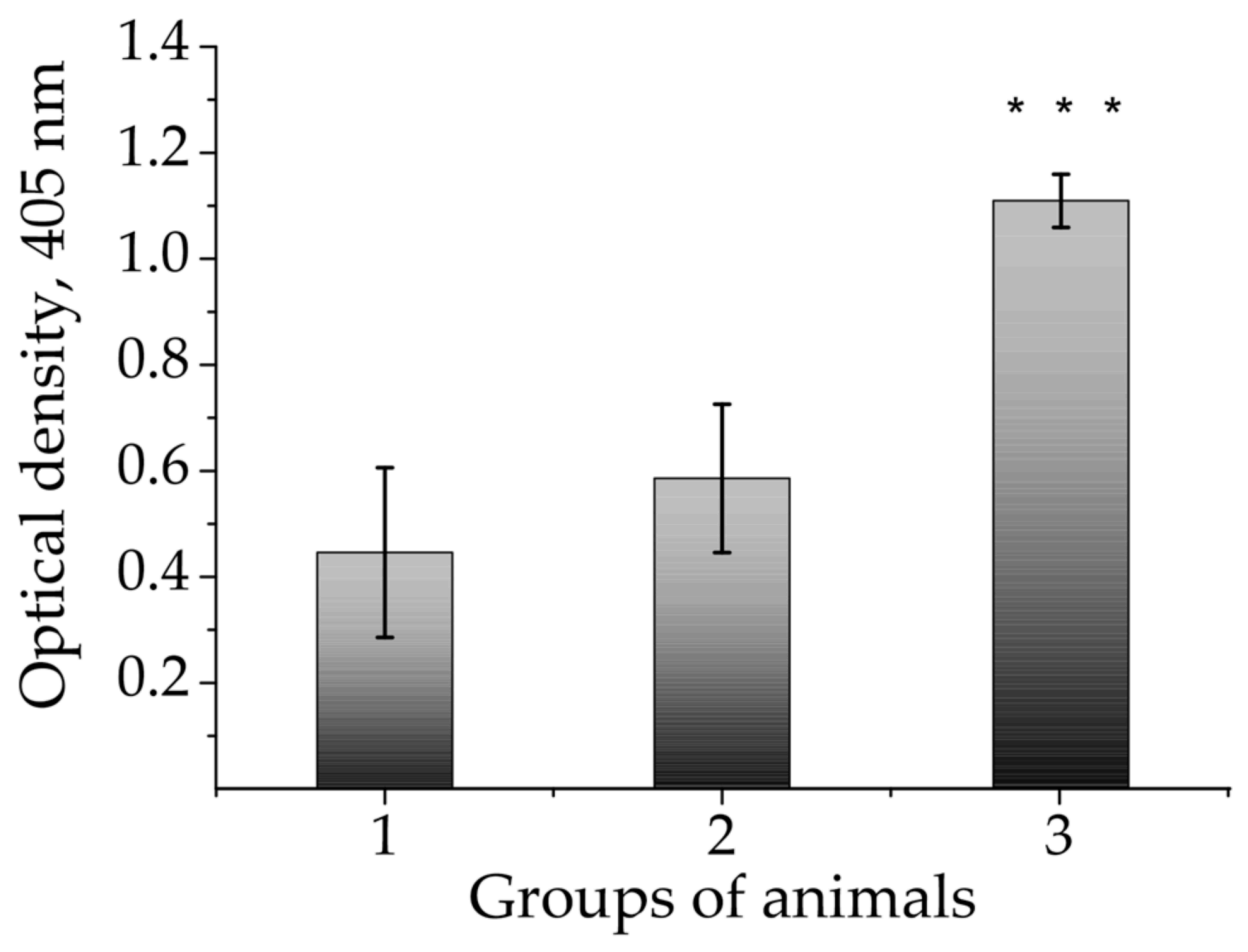
The level of anti-BSA antibodies in the blood serum of immunized mice: 1—control (PBS), 2—1st experimental group (BSA), 3—2nd experimental group (complex of BSA with the HG5 hydrogel). Statistically significant: *** *p* ≤ 0.001.

Polymeric adjuvants are a promising direction in vaccine development that combines nanotechnology, immunology, and materials science [55]. The advantage of using polymeric adjuvants includes the ability to increase the effectiveness of vaccines, especially for weak antigens; reduce the dose of antigen, which is important when the antigen is expensive or difficult to obtain; generate both cellular and humoral immune responses to protect against different types of pathogens; control the release of antigen; and provide longer-lasting protection. Only a limited number of adjuvants are approved and officially used in vaccines. The polymeric adjuvants themselves more often act as delivery systems, rather than as independently approved adjuvants. Among all synthetic biomaterials, polymeric hydrogels are the most similar to living tissues, as they have a high water content and a soft consistency similar to natural tissue. In addition, their biocompatibility facilitates their use in biological systems [56,57]. In immunology, adjuvants are used to increase or diversify the reaction to antigens. The hydrogel in this study was capable of presenting the antigen. This feature ensures an immune-specific response. White laboratory BALB/c mice were immunized subcutaneously with a model antigen, BSA. This protein was selected for its high solubility, well-defined structure, and biosafety, making it ideal for characterizing a new delivery platform (polymeric hydrogel adjuvant) before using complex pathogen antigens or expensive recombinant proteins. We observed a statistically significant increase in the level of specific antibodies against BSA in mice immunized with HG5 compared to both the control and the 1st experimental group (Figure 12).

Thus, the HG5 hydrogel possesses adjuvant properties and might be used in vaccine development.

The obtained results demonstrate that HG5 can effectively enhance immune responses and offers promise for vaccine formulation against infectious diseases. Moreover, HG5 may serve as an advanced vaccine adjuvant and delivery system due to its biocompatibility, low toxicity, and porous 3D network that enhances immune response.

## 3. Conclusions

Novel anionic cross-linked hydrogel microparticles (HG5) were successfully synthesized via precipitation polymerization of hydrophilic and hydrophobic monomers in the presence of a dimethacrylate cross-linker. Comprehensive physicochemical characterization by NMR, FT-IR, SAXS, TEM, and DLS confirmed the formation of spherical microparticles with a controlled hydrodynamic diameter of approximately 250 ± 50 nm and a narrow size distribution (PDI = 0.079). The ionized carboxyl groups imparted excellent aqueous dispersibility and colloidal stability at physiological pH (7.2–7.4), while enabling efficient incorporation of BSA as a model antigen. The incorporation of BSA was evidenced by the preservation of the particles’ hydrodynamic size, as determined by DLS and SAXS, together with changes in the internal structure reflected by an increase in the Porod exponent (β) from 1.71 to 2.48. HG5 exhibited a protein loading efficiency of 95%, while the hydrogel–protein complexes maintained their hydrodynamic size and colloidal stability for one month.

Biological evaluation demonstrated that HG5 exhibited low cytotoxicity toward pseudonormal cell lines (HaCaT keratinocytes, BJ fibroblasts, BEAS-2B bronchial epithelial cells, and HEK293 embryonic kidney cells), maintaining cell viability above 50% at concentrations up to 5 mg/mL. PBMCs were more sensitive to HG5 exposure, exhibiting an IC_50_ of 0.52 mg/mL. In addition, HG5 showed good hemocompatibility, with hemolysis below 5%, and did not induce pro-apoptotic morphological changes in HEK293 cells, including chromatin condensation, DNA fragmentation, or plasma membrane blebbing.

The ability of HG5 to form stable hydrogel–protein complexes translated into enhanced immunogenicity in vivo. Mice immunized with the HG5-based antigen formulation exhibited a 1.9-fold increase in BSA-specific antibody levels compared with immunization without an adjuvant (*p* < 0.05), demonstrating the adjuvant potential of the developed hydrogel system.

Overall, the developed HG5 microparticle system combines controlled physicochemical properties, efficient antigen loading, excellent colloidal stability, favorable biocompatibility, and the ability to enhance the humoral immune response to a model antigen. Although further studies using clinically relevant antigens and comprehensive evaluation of cellular immune responses are warranted, the present findings demonstrate that HG5 represents a promising hydrogel-based platform for protein delivery and vaccine adjuvant development, with potential applications in other biomedical fields.

## 4. Materials and Methods

### 4.1. Materials

The monomers of acrylic acid (AA, “Sigma-Aldrich”, St. Louis, MO, USA), glycidyl methacrylate (GMA, “Merck”, Darmstadt, Germany), and butyl acrylate (BA, “Merck”, Darmstadt, Germany) were used for polymer synthesis and purified by distillation under reduced pressure. Triethylene glycol dimethacrylate (TEGDMA), azobisisobutyronitrile (AIBN) as the radical initiator, and the solvents heptane, hexane, methanol, and ethanol were purchased from “Merck” (Darmstadt, Germany) and used as received without further purification. tert-Dodecylmercaptan (TDM) was supplied by “Sigma-Aldrich” (St. Louis, MO, USA) and used without further purification.

Hydrochloric and acetic acids, sodium hydroxide, and sodium chloride were purchased from “Fisher Scientific” (Hampton, NH, USA). Phosphate-buffered saline (PBS) was purchased from “Sigma-Aldrich” (St. Louis, MO, USA). Doxorubicin (Dox) was purchased from “Arterium” (Kyiv, Ukraine).

The following reagents were used for biological analysis: bovine serum albumin, BSA (molecular weight ~66 kDa); acrylamide/bis-acrylamide (29:1); N,N,N′, N′-tetramethylethylenediamine (TEMED); diphenylamine; sodium dodecyl sulfate (SDS); ammonium persulfate (APS); glycerol; and bromophenol blue (Laemmli buffer), all purchased from “Sigma-Aldrich” (St. Louis, MO, USA). β-mercaptoethanol (molecular biology grade) was obtained from “Millipore” (Darmstadt, Germany). Tris(hydroxymethyl)aminomethane hydrochloride was supplied by “Roche” (Basel, Switzerland). ELISA plates were obtained from “BioTechLab” (Kyiv, Ukraine). The fluorescent dyes 2′-(4-ethoxyphenyl)-5-(4-methyl-1-piperazinyl)-2,5′-bi-1H-benzimidazole trihydrochloride (Hoechst 33342) and 3,8-diamino-5-ethyl-6-phenylphenanthridinium bromide (ethidium bromide) were purchased from “Sigma-Aldrich” (St. Louis, MO, USA). A 1% solution of Crystal Violet (“Sigma-Aldrich”, St. Louis, MO, USA) was used in the clonogenic assay.

### 4.2. Synthesis

#### 4.2.1. Hydrogel Microparticles Synthesis

The principal methodological approaches of synthesis have been described previously [43,45]. However, in order to optimize the content of carboxylic groups and improve the stability of aqueous dispersions at physiological pH, optimized monomer compositions were used. Polymer hydrogel microparticles were synthesized using precipitation copolymerization of acrylic acid (AA), glycidyl methacrylate (GMA), butyl acrylate (BA), and triethylene glycol dimethacrylate (TEGDMA, all from “Sigma-Aldrich”, St. Louis, MO, USA), the latter serving as a cross-linking agent [58]. The characteristics of the obtained cross-linked hydrogel particles are presented in Table 1.

**Figure 13:**
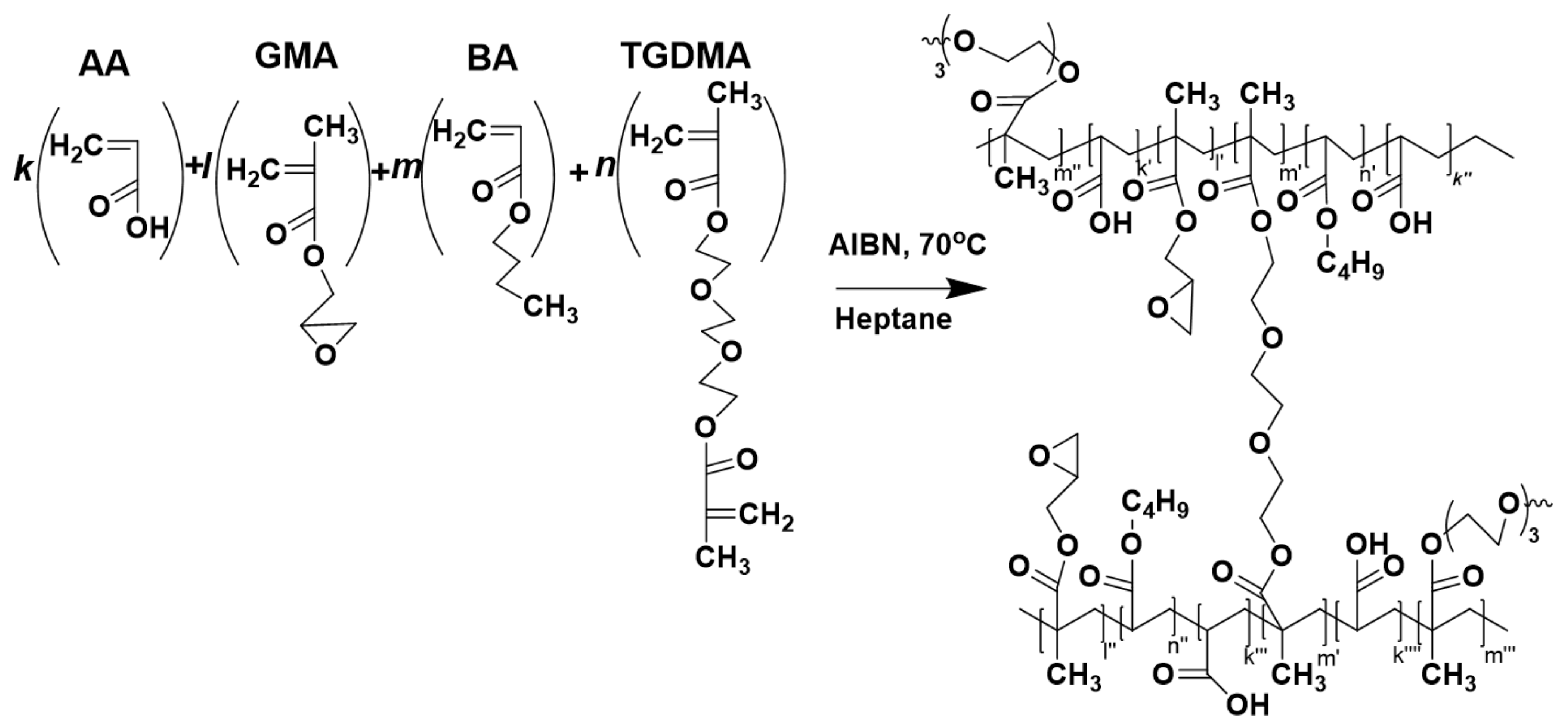
Schematic illustration of the hydrogel microparticles synthesis by radical precipitation copolymerization.

Hydrogel microparticles (HG5) were synthesized via precipitation copolymerization [59], as follows (Figure 13). AIBN (0.304 g, 1.85 mmol) was dissolved in a mixture of monomers comprising AA (1.638 mL, 22.73 mmol), GMA (0.29 mL, 2.03 mmol), BA (0.171 mL, 1.33 mmol), and TEGDMA (0.793 mL, 2.77 mmol). The total monomer concentration in the reaction mixture was 0.30 mol/L. Tert-dodecyl mercaptan (TDM) (0.023 mL, 0.1 mmol) was added to the mixture of monomers as a regulator of polymer chain length. The monomer–initiator mixture was purged with argon to remove dissolved oxygen, after which 97.11 mL of heptane was added, followed by a second argon purge. Polymerization was carried out in hermetically sealed 100 mL flat-bottom glass flasks under continuous magnetic stirring. Polymerization proceeded at 70 °C for 6 h under an argon atmosphere. The monomer conversion, determined gravimetrically [60], reached 95%. Residual monomers and soluble oligomeric species were removed by repeated washing of the hydrogel microparticles with ethanol, followed by centrifugation. The purified particles were then dried under vacuum at 60 °C until a constant weight was reached.

#### 4.2.2. Formation of Aqueous Dispersions

Water dispersions of hydrogel HG5 microparticles and their complexes with bovine serum albumin (BSA) were prepared as follows:

1. Preparation of HG5 dispersion. HG5 microparticles (0.4 g) were added to 9.0 mL of phosphate-buffered saline (PBS, pH 7.4) and stirred for 10 min to obtain a uniform dispersion. After dispersion, the system pH was approximately 3.0. The pH was adjusted to 7.4 by dropwise addition of 5 N NaOH under continuous stirring. The final volume was adjusted to 10 mL with PBS, if necessary. The dispersion was subsequently sonicated for 10 s.
2. Preparation of HG5–BSA complexes. HG5 microparticles (0.4 g) were dispersed in 5.0 mL of PBS. Separately, BSA (0.1 g) was dissolved in 4.0 mL of PBS. The BSA solution was added to the HG5 dispersion under constant stirring to allow complex formation. The pH was adjusted to 7.4 as described above. PBS was added to a final volume of 10 mL. The resulting dispersion was sonicated for 10 s.
3. HG5-BSA complex formation for stability studies. To evaluate antigen loading [61], HG5–BSA complexes were prepared by mixing equal volumes of 50 µg/mL HG5 and 25 µg/mL bovine serum albumin (BSA; “Sigma-Aldrich”, St. Louis, MO, USA) to a final volume of 2 mL. The mixture was incubated for 60–90 min at room temperature (RT) on an orbital shaker (120 rpm).

### 4.3. Product Characterization

#### 4.3.1. Hydrogel Microparticles

The functional composition of HG5, dispersed in deuterated water (D_2_O), was confirmed by ^1^H NMR spectroscopy [62]. Spectra were recorded on a Varian Mercury Plus 300 NMR spectrometer (“Varian Inc.”, Walnut Creek, CA, USA) at 300.13 MHz. The chemical structure of the HG5 was further verified by FT-IR spectroscopy using a Cary 630 FT-IR spectrometer (“Agilent Technologies”, Santa Clara, CA, USA). The chemical structure and functional group composition of the hydrogel microparticles were confirmed after synthesis, purification, and drying. The dried hydrogel microparticles were analyzed directly without any additional sample preparation (Figure S2A). For FTIR characterization of the HG5–BSA complex, the samples were prepared by lyophilization. Briefly, a dispersion of HG5 hydrogel particles (40 mg mL^−1^) and an HG5–BSA complex (HG5 = 40:4 mg mL^−1^) was prepared in PBS. The samples were subsequently freeze-dried according to the procedure described in [63]. The resulting lyophilized powders were analyzed directly by FTIR without redispersion (Figure S2B).

The carboxyl group content was determined by acid–base back titration, and the epoxy group content was determined by reverse titration of residual hydrochloric acid with 0.1 N NaOH. For determination of carboxyl groups, an excess of 0.1 N NaOH was added to a particle dispersion and stirred for 6 h at 60 °C. The insoluble gel particles were then washed with water and separated by centrifugation (Hettich Mikro 22R, “Andreas Hettich GmbH & Co. KG”, Tuttlingen, Germany) to prevent reagent sorption, and the total amount of the separated liquid phase containing residual NaOH was titrated with 0.1 N solution of HCl [43].

For determination of epoxy (EP) groups in the HG5, an excess of 0.1 N HCl in acetone was added to the particle dispersion and stirred for 6 h. The gel particles were washed and separated by centrifugation, and the total amount of the separated liquid phase containing residual HCl was titrated with 0.1 N NaOH [43].

Particle morphology was examined using a JEM-200A transmission electron microscope (“JEOL”, Tokyo, Japan) at an accelerating voltage of 200 kV. Samples from aqueous or organic dispersions were prepared at a particle concentration of 4 mg/mL in bi-distilled water or toluene. Specimens were deposited onto substrates by ultrasonic spraying using a UZDN-1A disperser (“UkrRosPribor Ltd.”, Kyiv, Ukraine), forming a uniform coating. A thin amorphous carbon film on a copper grid served as the supporting surface [64].

#### 4.3.2. Swelling Analysis of HG5 Hydrogel

Swelling experiments for the HG5 gel were conducted as follows [65,66]. Initially, the dry polymer powder was placed into a nylon mesh bag and immersed in a distilled water bath until equilibrium swelling was achieved (approximately 24 h). After equilibration, the swollen gel was transferred onto a glass slide and kept for 2–3 min to remove excess water. The gel samples were then placed into two separate cuvettes of identical and known volume (V_0_ = 0.105 ± 0.005 cm^3^). The cuvettes containing the gel samples were dried in a chamber at 40 °C for 48 h. The weights of the samples before and after drying were recorded. Upon complete drying, the gel volume decreased; therefore, isopropyl alcohol was added dropwise until the initial volume V_0_ was restored. The weight of the added isopropyl alcohol m1 was measured, and its volume V_1_ was determined using the density of isopropyl alcohol (ρ_1_ = 0.786 ± 0.008 g/cm^3^). The volume of the dry gel was then calculated as V_dry_ = V_0_ − V_1_. The swelling coefficient Q was calculated as Q = V_0_/V_dry_ [67]. The isotropic expansion factor α was determined from α = Q^1/3^. Additionally, the densities of the dry (ρ_dry_ = 1.5 ± 0.06 g/cm^3^) and swollen (ρ_swollen_ = 1.01 ± 0.01 g/cm^3^) gels were calculated from the measured weights and corresponding volumes [68].

#### 4.3.3. Efficiency of BSA Loading

Efficiency of BSA loading was determined using a bicinchoninic acid (BCA) assay (BCA Protein Quantitation Kit, “Interchim”, Montluçon, France). HG5 and BSA were dissolved in 0.1 M NaCl (in deionized water) at a 2:1 mass ratio (HG5-BSA) and incubated for 60 min at RT on a shaker (120 rpm) [69]. Then, samples were centrifuged at 12,000× *g* for 6 min. A 100 µL aliquot of supernatant was mixed with 2 mL of BCA reagents, incubated at 60 °C for 30 min, cooled to RT, and the absorbance was measured at 562 nm.

#### 4.3.4. Dynamic Light Scattering (DLS)

The particle size of the HG5 hydrogel was determined using dynamic light scattering (DLS) with a Litesizer DLS 500 instrument (Anton Paar, Graz, Austria). Prior to measurement, each solution was sonicated in a water bath for 20–30 min at 20–50 °C. All measurements were performed at a constant temperature of 25 °C following a 3 min equilibration time for each sample. To determine the optimal dispersion conditions, stock solutions of HG5 were prepared in a 0.154 M NaCl-dH_2_O solution (pH 7.4) at varying concentrations (5, 2, 1, 0.5, and 0.1 mg/mL). To ensure data reliability, each measurement was performed in five replicates, and the reading demonstrating the lowest polydispersity index (PDI) was recorded. The concentration yielding the most reproducible and stable measurements was selected for all subsequent analyses.

Zeta potential was also measured using the NanoPartica SZ-100 (“Horiba, Ltd.”, Kyoto, Japan) at 25 °C. Sample concentrations were 0.4 mg/mL (HG5) and 0.1 mg/mL (BSA). Three to five measurements were performed per sample, each consisting of five runs with 5 min intervals between measurements.

#### 4.3.5. Small-Angle X-Ray Scattering (SAXS)

Small-angle X-ray scattering (SAXS) measurement was conducted at the P12 BioSAXS beamline [70] of the European Molecular Biology Laboratory (EMBL) at the PETRA III storage ring of the Deutsche Elektronen Synchrotron (DESY, Hamburg, Germany). Data were collected using a Pilatus 6M detector (“Dectris”, Würenlingen, Switzerland) with synchrotron radiation of wavelength λ = 1.2 Å. A sample-to-detector distance of 3 m enabled measurements over a momentum transfer range of 0.02 to 4 nm^−1^. The q-range calibration was performed using the diffraction pattern of silver behenate.

Scattering intensities were normalized to the transmitted beam intensity, and background scattering from the aqueous buffer was subtracted. Aliquots of 40 μL of each sample and corresponding buffer were loaded into the measurement cell using an automated sample changer (“Arinax”, Moirans, France, BioSAXS). The temperature was maintained at 37.0 ± 0.2 °C for both samples and the buffer. For each measurement, 40 consecutive frames with an exposure time of 0.095 s per frame were recorded for both sample and solvent.

To exclude radiation-induced artifacts, individual scattering curves within each dataset were compared with a reference curve (typically the first exposure). The validated data were subsequently averaged using the automated data acquisition and processing pipeline described by Franke et al. [71].

#### 4.3.6. SDS-PAGE Electrophoresis and Coomassie Brilliant Blue Staining

Complex formation was confirmed by SDS-PAGE followed by Coomassie Brilliant Blue staining. A resolving gel (12% acrylamide/bis-acrylamide, 29:1) was prepared using 0.375 M Tris-HCl (pH 8.8), 0.1% SDS, 0.1% ammonium persulfate (APS), and 0.001% TEMED. A 4% stacking gel containing 0.125 M Tris-HCl (pH 6.8), 0.1% SDS, 0.1% APS, and 0.001% TEMED was overlaid. Electrophoresis was carried out in running buffer (25 mM Tris, 192 mM glycine, 0.1% SDS) at 80 V (stacking) and 120 V (resolving). Samples included positive controls (50 µg BSA), a negative control (50 µg HG5), and HG5–BSA complex samples (60 µL each). All were incubated at RT for 30 min, mixed with 5× Laemmli buffer (0.125 M Tris-HCl, pH 6.8, 1% SDS, 10% glycerol, 0.01%, 5% β-mercaptoethanol), and denatured at 95 °C for 5 min. Twenty-five microliters of each sample were loaded per lane. Following electrophoresis, gels were stained with 0.25% Coomassie Brilliant Blue R-250 in 50% methanol, 10% acetic acid, and 40% deionized water at 37 °C and then destained with 50% methanol and 10% acetic acid until bands were clearly visible [72]. The intensity of the BSA bands was quantified by densitometric analysis using ImageJ software (version 1.53k, National Institutes of Health, Bethesda, MD, USA). Band intensities were quantified using the “Plot Lanes” function, and the area under the curve (AUC) of each BSA band peak was calculated. Relative band intensities were obtained by normalization to the free BSA control (set as 100%). The densitometric analysis was performed on a single representative SDS-PAGE gel (*n* = 1), as the experiment was not repeated under identical experimental conditions.

#### 4.3.7. Refractive Index Analysis

Hydrogel solutions were prepared in phosphate-buffered saline (PBS) for refractive index (RI) determination. To ensure complete dissolution, samples were subjected to bath sonication at 20–50 °C for 20–30 min. RI measurements were performed using a Litesizer DLS 500 instrument (“Anton Paar”, Graz, Austria), following the manufacturer’s “RI Analysis” protocol.

#### 4.3.8. Measurement of Stability of HG5-BSA Complex

The stability of the HG5-BSA complex (mass ratio equaled 1:2.5) was evaluated over a period of four weeks at RT [73]. As previously reported, nanogel systems and particle size monitoring by DLS were conducted to assess structural stability and aggregation behavior over time [74]. Particle size measurements were taken at predetermined time points, with five replicates per measurement, and the average values were recorded.

The stability of aqueous dispersions was evaluated by monitoring turbidity over time using a Turboquant^®^ 1500 Turbidimeter (“Merck”, Darmstadt, Germany).

#### 4.3.9. Storage Stability Assessment of HG5–BSA Complexes

Storage stability and BSA retention behavior of the HG5–BSA complex were evaluated over a one-month period. HG5–BSA complexes (mass ratio 2:1) were prepared in PBS and immediately divided into single-use aliquots of 2 mL to avoid repeated freeze–thaw cycles, with three independent aliquots prepared for each storage condition. The aliquoted samples were stored at −20 °C, 4 °C, and 22 °C for up to 30 days. At each storage condition, a fresh, previously unsampled aliquot was withdrawn on days 1, 3, 5, 7, 14, 21, and 30 for analysis. Each withdrawn aliquot was centrifuged at 14,000× *g* at 4 °C for 10 min before BSA quantification. The BSA content in the resulting supernatant was quantified using the BCA assay, following the procedure described in Section 4.3.3, with absorbance measured at 562 nm using an SP-UV 300SRB spectrophotometer (Spectrum Instruments, Shanghai, China). The measured BSA concentration in the supernatant was used to estimate the fraction of BSA retained within or associated with the HG5–BSA complex at each time point according to the following equation: % BSA retention = (1 − [BSA]supernatant/[BSA]total) × 100. This approach enabled the assessment of BSA retention and the storage stability of the HG5–BSA complex under the three investigated temperature conditions [75].

### 4.4. Biological Evaluation

#### 4.4.1. Hemolytic Activity

Hemolytic activity was assessed using fresh human blood collected from healthy human donors after obtaining written informed consent. Blood was centrifuged at 3000 rpm for 5 min to isolate erythrocytes, which were washed twice with PBS (pH 7.4) and resuspended to the original volume. HG5 solutions (2.5 mg/mL and 0.25 mg/mL) were incubated with erythrocytes at 37 °C with shaking (300 rpm) for 1.5 h [76]. PBS served as a negative control and 0.1% Triton X-100 as a positive control. After incubation, samples were centrifuged (3000 rpm, 5 min, 4 °C), and the absorbance of the supernatant was measured at 540 nm to quantify hemoglobin release. Both absorbance readings and visual inspection were used to evaluate hemolysis.

#### 4.4.2. Immunological Evaluation

An indirect ELISA was applied to assess the immunological efficiency of the studied hydrogel particles (HG5). 100 μL of 1% BSA solution was adsorbed onto a 96-well plate (PAA, Pasching, Austria) and incubated for 24 h at 4 °C. Then, the wells were washed three times with buffer A (0.2% BSA in PBS), and 10 µL of blood serum in PBS was added to the wells and incubated for 2 h at 37 °C, rinsed three times with buffer A, and conjugated anti-mouse antibodies (“Abcam”, Cambridge, MA, USA) were added in a 1:5000 dilution and incubated for 1 h at 37 °C. The wells were washed three times with PBS-Tween-20, and the substrate for alkaline phosphatase, p-nitrophenylphosphate in diethanolamine, was added. After incubating at room temperature for 3 min [43], absorbance was measured at 405 nm on an ELISA plate reader (“STAT FAX Awareness Technology Inc.”, Palm, FL, USA).

#### 4.4.3. Cell Culture

Human chronic myelogenous leukemia K562 (ATCC: CCL-243) cells, human keratinocytes of the HaCaT (RRID: CVCL_0038) line, and human embryonic kidney HEK293 (ATCC: CRL-1573) cells were obtained from the Cell Collection of R.E. Kavetsky Institute of Experimental Pathology, Oncology and Radiobiology (Kyiv, Ukraine). Human acute leukemia Jurkat (ATCC: TIB-152) T-cells were obtained from the Institute of Cancer Research, Medical University of Vienna (Vienna, Austria). Human fibroblasts of the BJ (ATCC: CRL-2522) line and human colorectal carcinoma HT-29 (ATCC: HTB-38) cells were obtained from the Medical University of Bialystok (Białystok, Poland). Human bronchial epithelial cells of the BEAS-2B (ATCC: CRL-9609) line were obtained from the Institute Gustave Roussy (Villejuif, France).

Cells were cultured in DMEM, RPMI-1640, or McCoy’s 5A Modified Medium supplemented with 10% fetal bovine serum and 1% Penicillin-Streptomycin (all from “Sigma-Aldrich”, St. Louis, MO, USA). Cells were incubated in a humidified CO_2_ incubator at 37 °C.

Human PBMCs were isolated from a healthy adult donor’s peripheral blood containing an anticoagulant sodium heparin solution (10 U/mL) using a density gradient of Gradisol G (Polfa, Warsaw, Poland), as reported previously [77].

The Bioethics Committee of the Institute of Cell Biology of the National Academy of Sciences of Ukraine authorized the study using PBMCs isolated from a healthy adult human donor (Protocol No. 2024-1 from 13 February 2024, and Protocol No. 2025-1 from 5 February 2025) after the donor provided written consent. The PBMCs were grown in the RPMI-1640 medium containing 20% fetal bovine serum and 1% Penicillin-Streptomycin.

#### 4.4.4. MTT Assay

Cytotoxicity of HG5 and doxorubicin (reference standard) in PBMCs, pseudonormal, and tumor cell lines was determined in vitro via the MTT assay, following the protocol described previously [78]. Mammalian cells growing in a monolayer were placed in 96-well plates (5 × 10^3^ cells/well, 100 μL) and left to attach for 24 h. Suspension mammalian cells were plated at 1.5 × 10^4^ cells/100 μL per well. PBMCs were plated at 1 × 10^5^ cells/100 μL per well. Following seeding, cells were exposed to graded concentrations (0–5 mg/mL) of the test hydrogel for 72 h. Subsequently, MTT solution was added, and the resultant formazan crystals were solubilized in DMSO (“Sigma-Aldrich”, St. Louis, MO, USA). Optical density was quantified using a BioTek ELx800 microplate reader (“BioTek Instruments, Inc.”, Winooski, VT, USA).

#### 4.4.5. Cell Colony-Forming Assay

HaCaT cells (500 cells/well) were dispensed into 12-well plates containing 2 mL of culture medium and allowed to attach overnight. The cells were then exposed to HG5 across a concentration range of 0.1–10 mg/mL. After 14 days, cell colonies were fixed using ice-cold methanol (10 min, 4 °C) and subsequently stained with 0.1% crystal violet (“Sigma-Aldrich”, St. Louis, MO, USA) for 10 min, rinsed, and air-dried at room temperature [79]. Colony numbers were determined with ImageJ software (version 1.44p; National Institutes of Health, Bethesda, MD, USA), and results were presented as the percentage of colonies relative to untreated controls.

#### 4.4.6. Fluorescence Microscopy of Cells

HEK293 cells were seeded onto glass slides in 12-well plates at 5 × 10^4^ cells/well in complete growth medium. Following adhesion, cells were subsequently exposed to HG5 at a concentration of 5 mg/mL for 72 h.

Hoechst 33242 fluorescent dye was added directly to the culture medium at a final concentration of 1–2 μg/mL (“Sigma-Aldrich”, St. Louis, MO, USA) and incubated for 15 to 30 min at 37 °C in a CO_2_ incubator. Excess dye was removed by washing twice with pre-warmed Hank’s Balanced Salt Solution (HBSS, pH 7.4) (“Thermo Fisher Scientific”, Waltham, MA, USA) to minimize background fluorescence.

Rhodamine 123 (200–500 nM final concentration; “Sigma-Aldrich”, St. Louis, MO, USA) was added directly to the culture medium, and cells were incubated for 20–30 min, protected from light. Unincorporated dye was cleared by performing two washes with pre-warmed HBSS.

Live-cell imaging was performed under a Zeiss AxioImager A1 fluorescence microscope fitted with an A1 camera and specialized imaging software (”Carl Zeiss”, Oberkochen, Germany) with a ×40 objective and appropriate filter sets (Hoechst: excitation 350/50 nm, emission 460/50 nm; Rhodamine 123: excitation 510–550 nm, emission 570–620 nm). Images were additionally analyzed using Image-Pro Premier 7 software (“Media Cybernetics”, Rockville, MD, USA).

#### 4.4.7. Diphenylamine Assay of DNA Fragmentation

HEK293 cells were seeded at a density of 5 × 10^5^ cells/well in 12-well plates and allowed to adhere overnight. Cells were subsequently treated with HG5 at a concentration of 5 mg/mL for 72 h. Following treatment, cells were lysed, and genomic DNA was extracted according to the established protocol [80]. Samples were processed with 1.5% diphenylamine (“Sigma-Aldrich”, St. Louis, MO, USA), and absorbance readings were obtained at 630 nm with a microplate reader BioTek ELx800 (“BioTek Instruments”, Winooski, VT, USA). The percentage of fragmented DNA was calculated using the formula: [OD(B)/(OD(A) + OD(B))] × 100, where OD(A) represents the absorbance of the non-fragmented DNA fraction and OD(B) represents the absorbance of fragmented DNA fractions.

#### 4.4.8. Alkaline Comet Assay

HEK293 cells were exposed to HG5 (5 mg/mL) for 72 h. DNA damage was evaluated using the comet assay under alkaline conditions, as previously described [81]. After electrophoresis, slides were stained with ethidium bromide (10 μg/mL; “Sigma-Aldrich”, St. Louis, MO, USA) and imaged under Zeiss AxioImager A1 fluorescence microscope fitted with an A1 camera and specialized imaging software (”Carl Zeiss”, Oberkochen, Germany). DNA damage was quantitatively evaluated with CASPLab software (version 1.2.3b2; CASPLab, Wrocław, Poland), employing the percentage of DNA in the tail (%Tail) as the principal parameter for DNA damage identification.

#### 4.4.9. Statistical Analysis

Data are expressed as mean ± standard deviation (M ± SD, *n* = 4). GraphPad Prism software (version 8 or 9; GraphPad Software, San Diego, CA, USA) was used for data plots and statistical analysis. Differences between groups were assessed by ANOVA, followed by Dunnett’s multiple comparisons test for the in vitro and in vivo studies, with statistical significance at *p* < 0.05. For the remaining experiments, one-way ANOVA followed by Tukey’s post hoc Test was employed, applying the same significance threshold (*p* < 0.05).

## Figures and Tables

**Table 1:** Compositions and some colloidal-chemical characteristics of HG5 particles (pH 7.4).

Sample	Composition of Hydrogel Copolymer Particles, % mol				BSA Content in the Complex, g/100 g HG5	D_h_, nm	Z-Potential, mV
	AA Unit	GMA Unit	BA Unit	TGDMA Unit			
HG5	78	7	4	11	-	250 ± 50	−15.0 ± 2.5
Complex HG5-BSA					95	250 ± 55	−7.0 ± 1.2

## Data Availability

Data are contained within the article or Supplementary Materials.

## Supplementary Materials

The following supporting information can be downloaded at: https://www.mdpi.com/article/10.3390/gels12080677/s1, Figure S1: ^1^H NMR spectra of HG5 hydrogel particles; Figure S2: FTIR spectra of (A) dry HG5 hydrogel particles and (B) albumin (BSA) and lyophilized HG5 and complexes HG5-BSA; Figure S3: Loading efficiency of HG5-BSA complex at 22 °C. 562 nm absorbance values of negative control (BSA + 0.154 M NaCl-dH_2_O), positive control (HG5 + 0.154 M NaCl-dH_2_O), and HG5 + BSA mixture; Figure S4: The viability of human Jurkat T-cell leukemia, chronic myeloid leukemia K562 cells, and colorectal carcinoma HT-29 cells treated for 72 h with HG5 hydrogel. Results of MTT assay are expressed as a percentage of the control group and presented as M ± SD, *n* = 4. * *p* < 0.05; *** *p* < 0.001 compared to control (non-treated) cells; Figure S5: Quantitative identification of DNA fragmentation after 72 h of HG5 treatment via the diphenylamine method. Data are expressed as M ± SD, *n* = 4; Figure S6: The results of the DNA comet assay in alkaline conditions for the detection of DNA damage in HEK293 cells treated for 72 h with HG5 at a 5 mg/mL dose: A—representative photographs of comets of control (non-treated) cells; B—the percentage of tail DNA in treated HEK293 cells. Table S1: Structural parameters calculated for hydrogels and their complexes with the BSA extracted from SAXS profiles using the Guinier, Porod, and GNOM IFT fitting procedures.
