# Supplementary material for: Bioinspired Cross-Linked Anionic Hydrogel Microparticles as Potential Adjuvants for Safe Vaccines: Colloidal-Chemical Characteristics, Morphology and Biological Properties
Source: Gels. 2026 Jul 31;12(8):677. doi: 10.3390/gels12080677 (PMC13511896; doi:10.3390/gels12080677)
Supplement: Supplementary file 1 [file gels-12-00677-s001.zip › gels-4442143-supplementary.pdf]

Supplementary Materials for

Bioinspired cross-linked anionic hydrogel microparticles as potential adjuvants for safe vaccines: colloidal-chemical characteristics, morphology and biological properties

Sebnem Ercelen Ceylan[1*†], Nataliya Mitina[2], Nataliya Finiuk[3], Bunyamin Bulkurcuoglu[4], Saygin San[1], Aise Rumeysa Mazi[1], Mariya Kozak[5,6], Oleh Izhyk[2,7], Iryna Petruh[5], Vasil M. Garamus[8], Khrystyna Harhay[2], Roman Nebesnyi[2], Rostyslav Stoika[3], Alexander Zaichenko[2,5*†]

[1] University of Health Sciences, Hamidiye Faculty of Medicine, Department of Biophysics, Selimiye Mah. Tıbbiye Cad.38, 34668, Istanbul, Türkiye; sebnem.ercelenceylan@sbu.edu.tr (S.E.C.); saygin.san@sbu.edu.tr (S.S); aiserumeysa.mazi@sbu.edu.tr (A.R.M.);

[2] Lviv Polytechnic National University, Institute of Chemistry, Department of Organic Chemistry, 12, Stepan Bandera Str, 79013, Lviv, Ukraine; nataliia.y.mitina@lpnu.ua (N.M.); oleh.b.izhyk@lpnu.ua (O.I.); khrystyna.i.harhai@lpnu.ua (Kh.H.); roman.v.nebesnyi@lpnu.ua (R.N.); oleksandr.s.zaichenko@lpnu.ua (A.Z.);

[3] Institute of Cell Biology of the National Academy of Sciences of Ukraine, Department of Regulation of Cell Proliferation and Apoptosis, 16, Drahomanov Str., 79005, Lviv, Ukraine; nataliyafiniuk@gmail.com (N.F.); stoika.rostyslav@gmail.com (R.S.);

[4] İstanbul Health and Science University, Faculty of Medicine, Department of Medical Biology, Sütlüce Mah. İmrahor Cad.82 34445, Istanbul, Türkiye; bunyamin.bulkurcuoglu@istun.edu.tr (B.B.)

[5] Institute of Animal Biology NAAS Ukraine, Laboratory of Molecular Biology and Genetics, 38, V. Stus str., Lviv, 79034, Ukraine; mariyarkozak@gmail.com; irapetruh@ukr.net (I.P.)

[6] Stepan Gzhytskyi National University of Veterinary Medicine and Biotechnologies, Faculty of Veterinary Medicine, Pekarska St, 50, Lviv, 79000, Ukraine; mariyarkozak@gmail.com (M.K.)

[7] Ivan Franko National University of Lviv, Department of Optoelectronics and Information Technologies, 1, Universytetska St., 79000, Lviv, Ukraine; oleh.b.izhyk@lpnu.ua (O.I.)

[8] Helmholtz Zentrum Hereon, Max-Planck-Str. 1, 21502 Geesthacht, Germany; vasyl.haramus@hereon.de (V.G.)

* Correspondence: sebnem.ercelenceylan@sbu.edu.tr (S.E.C.); oleksandr.s.zaichenko@lpnu.ua (A.Z.)

† These authors contributed equally to this work.

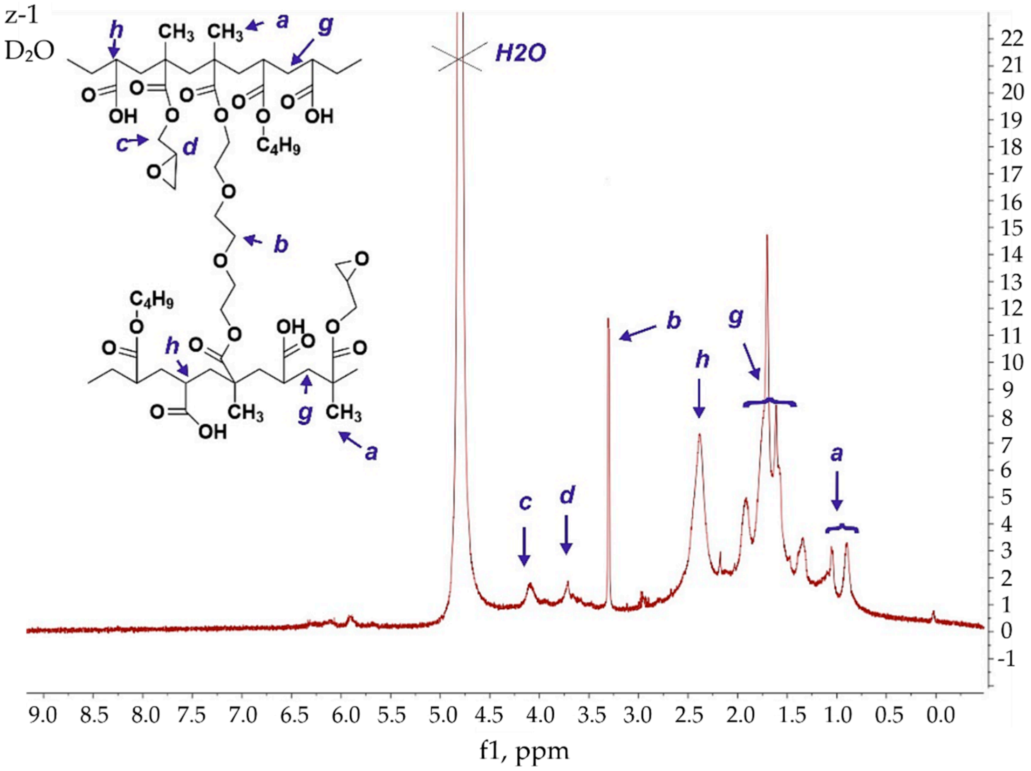

Figure S1. ^{1}H NMR spectra of HG5 hydrogel particles.

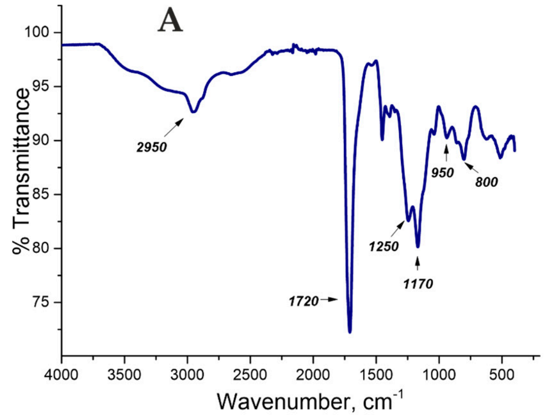

Wave (cm^{-1})	Identification of absorption bands
2950-2870	ν_{as} -CH_2-; -CH- (polymer backbone)
1720	ν -C=O (for all acrylates)
sets of peaks 1498-1429	δ_{as} CH_3; scissor vibr. CH_2
1357	δ_{sim}–$C(CH)_3$
1250	ν_{as} -C-O-C-, oxiran (GMA)
1170	ν_{as} -C-O-C-, aliphatic ether (TGDMA)
950	out-of-plane bending -OH, intermolecular interaction of carboxyl groups AA
800	δ_{as} C-H

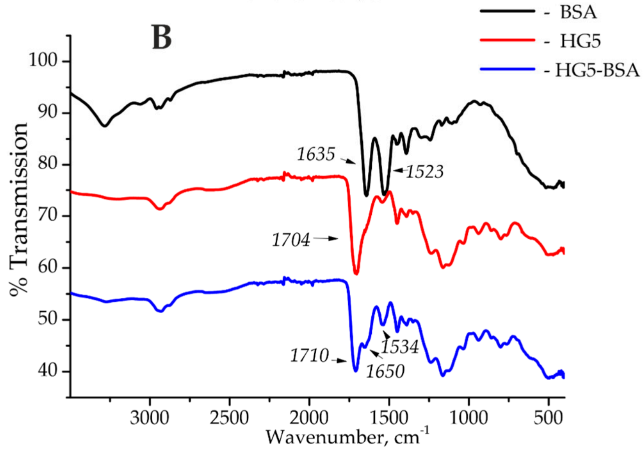

Wave (cm-1)	Identification of absorption bands
1635 cm^{-1}	NH_2 scissoring vibration of the N-terminal amino groups of BSA.
1523cm^{-1}	N–H bending (amide II) vibration of BSA peptide fragments
1704 cm^{-1}	C=O stretching vibration of the carbonyl groups in the acrylate units of HG5, including acrylic acid moieties.
1710 cm^{-1}.	C=O stretching vibration of the carbonyl groups in the acrylate units of HG5; the shift is attributed to the formation of the HG5–BSA complex
1650 cm^{-1} and 1534 cm^{-1}	Amide I and amide II bands of BSA, respectively; the observed shifts are attributed to the formation of the HG5–BSA complex.

Figure S2. FTIR spectra of A) dry HG5 hydrogel particles and B) albumin (BSA) and lyophilized HG5 and complexes HG5-BSA.

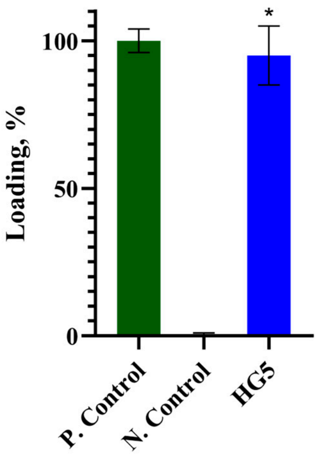

Figure S3. Loading efficiency of HG5-BSA complex at 22 °C. 562 nm absorbance values of negative control (BSA+0.154 m NaCl-dH_2O), positive control (HG5+0.154 m NaCl-dH_2O) and HG5+BSA mixture.

Table S1. Structural parameters calculated for hydrogels and their complexes with the BSA extracted from SAXS profiles using Guinier, Porod and GNOM IFT fitting procedure.

Sample	R_{g1} (Guinier), nm	R_{g2} (IFT), nm	D_{max}, nm	Scaling law at low q, α	Porod exponent, β
HG5	80.6±3.0	90.8±7.0	290±10	3.52±0.02	1.71±0.02
HG5-BSA	80.9±3.0	90.2±9.0	285±10	3.54±0.02	2.48±0.02

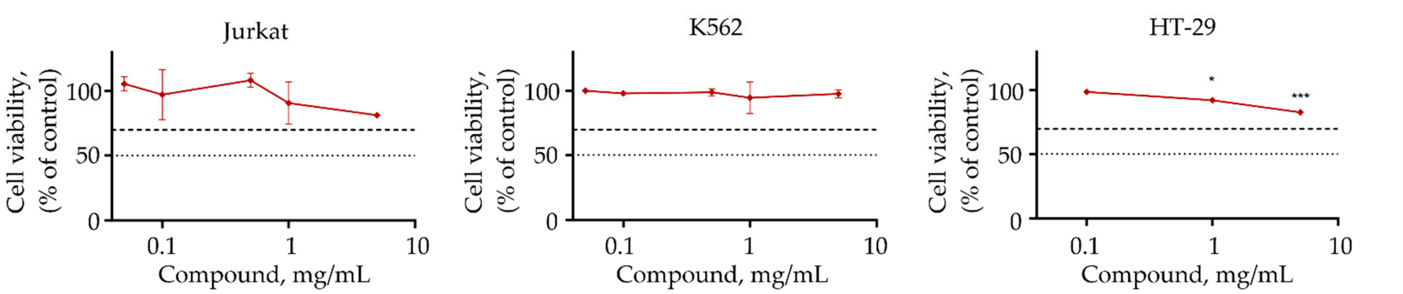

Figure S4. The viability of human Jurkat T-cell leukemia, chronic myeloid leukemia K562 cells, and colorectal carcinoma HT-29 cells treated for 72 hours with HG5 hydrogel. Results of MTT assay are expressed as a percentage from the control group and presented as M ± SD, n=4. **P < 0.05; ***P < 0.001 compared to control (non-treated) cells.*

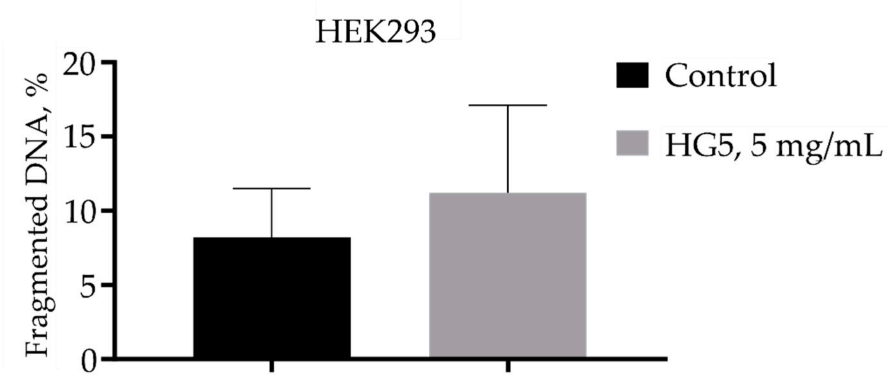

Figure S5. Quantitative identification of DNA fragmentation after 72 h of HG5 treatment using the diphenylamine method. Data are expressed as M± SD, n=4.

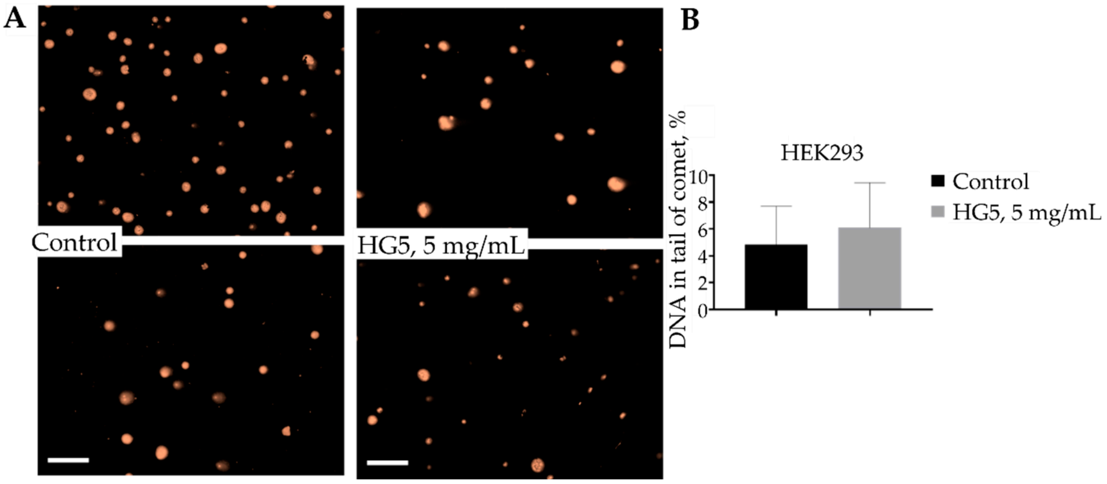

Figure S6. The results of DNA comet assay in alkaline conditions for detection of DNA damage in HEK293 cells treated for 72 h with the HG5 at 5 mg/mL dose: A – representative photographs of comets of control (not-treated) cells; B – the percentage of tail DNA in treated HEK293 cells.
